# Modular self-assembly system for development of oligomeric, highly internalizing and potent cytotoxic conjugates targeting fibroblast growth factor receptors

**DOI:** 10.1186/s12929-021-00767-x

**Published:** 2021-10-11

**Authors:** Marta Poźniak, Natalia Porębska, Kamil Jastrzębski, Mateusz Adam Krzyścik, Marika Kucińska, Weronika Zarzycka, Agnieszka Barbach, Małgorzata Zakrzewska, Jacek Otlewski, Marta Miączyńska, Łukasz Opaliński

**Affiliations:** 1 Faculty of Biotechnology, Department of Protein Engineering, University of Wroclaw, Joliot-Curie 14a, 50-383 Wroclaw, Poland; 2 Laboratory of Cell Biology, International Institute of Molecular and Cell Biology, 02-109 Warsaw, Poland

**Keywords:** FGF/FGFR, Endocytosis, Cancer, Targeted therapy

## Abstract

**Background:**

Overexpression of FGFR1 is observed in numerous tumors and therefore this receptor constitutes an attractive molecular target for selective cancer treatment with cytotoxic conjugates. The success of cancer therapy with cytotoxic conjugates largely relies on the precise recognition of a cancer-specific marker by a targeting molecule within the conjugate and its subsequent cellular internalization by receptor mediated endocytosis. We have recently demonstrated that efficiency and mechanism of FGFR1 internalization are governed by spatial distribution of the receptor in the plasma membrane, where clustering of FGFR1 into larger oligomers stimulated fast and highly efficient uptake of the receptor by simultaneous engagement of multiple endocytic routes. Based on these findings we aimed to develop a modular, self-assembly system for generation of oligomeric cytotoxic conjugates, capable of FGFR1 clustering, for targeting FGFR1-overproducing cancer cells.

**Methods:**

Engineered FGF1 was used as FGFR1-recognition molecule and tailored for enhanced stability and site-specific attachment of the cytotoxic drug. Modified streptavidin, allowing for controlled oligomerization of FGF1 variant was used for self-assembly of well-defined FGF1 oligomers of different valency and oligomeric cytotoxic conjugate. Protein biochemistry methods were applied to obtain highly pure FGF1 oligomers and the oligomeric cytotoxic conjugate. Diverse biophysical, biochemical and cell biology tests were used to evaluate FGFR1 binding, internalization and the cytotoxicity of obtained oligomers.

**Results:**

Developed multivalent FGF1 complexes are characterized by well-defined architecture, enhanced FGFR1 binding and improved cellular uptake. This successful strategy was applied to construct tetrameric cytotoxic conjugate targeting FGFR1-producing cancer cells. We have shown that enhanced affinity for the receptor and improved internalization result in a superior cytotoxicity of the tetrameric conjugate compared to the monomeric one.

**Conclusions:**

Our data implicate that oligomerization of the targeting molecules constitutes an attractive strategy for improvement of the cytotoxicity of conjugates recognizing cancer-specific biomarkers. Importantly, the presented approach can be easily adapted for other tumor markers.

**Supplementary Information:**

The online version contains supplementary material available at 10.1186/s12929-021-00767-x.

## Background

Cancers is one of the leading causes of death worldwide, and the number of affected people is constantly growing. Currently, conventional cancer treatment methods include surgical intervention, radiotherapy, and chemotherapy. These approaches, although successful to some extent, demonstrate low specificity against tumor cells and may evoke numerous side effects [1]. Therefore, methods for precise elimination of the tumor cells are urgently needed. This goal can be achieved by employing precision medicine, where therapeutic modalities are based on specific molecular characteristics of a patient's tumor [2, 3]. Every cancer cell is equipped with a wide range of biologically active surface molecules, which include MHC or HLA antigens, cytokine receptors, cell-adhesion molecules, growth factor receptors, Fas/Fas-ligand molecules and others. The expression of these biomarkers is upregulated in cancers facilitating tumor growth and spread [3–5]. On the other hand, tumor-overproduced biomarkers, which are produced at very low level or not at all by the healthy cells, constitute very attractive molecular targets for development of selective therapies [6, 7]. In the targeted anti-cancer approaches tumor-specific biomarkers facilitate precise delivery of cytotoxic payload into cancer cells, bypassing the healthy ones. Various drug delivery agents, including monoclonal antibodies, antibody fragments and receptor ligands have been covalently conjugated with potent cytotoxic drugs and applied for cancer treatment [8–10]. The precise recognition of a cancer biomarker by a targeting molecule within a cytotoxic conjugate and ability of the targeting molecule to enter the cancer cell interior by receptor-mediated endocytosis constitute key steps in the targeted anti-cancer therapies [11–13].

Fibroblast growth factor receptor 1 (FGFR1) is a receptor tyrosine kinase (RTK) that cooperates with the extracellular fibroblast growth factors (FGFs) in promoting cell proliferation, migration, differentiation and apoptosis [14, 15]. Elevated level of FGFR1 has been observed in various tumor types, like breast, lung, head or neck cancers [16–21]. Thus, FGFR1 is considered as an attractive tumor biomarker and several approaches for selective treatment of FGFR1 overproducing cancer cells have been developed, including cytotoxic conjugates with antibody fragments or natural ligands as targeting agents [22–25]. We have recently shown that the spatial distribution of FGFR1 at the plasma membrane determines efficiency and mechanism of receptor endocytosis [25]. Dimerization of FGFR1 with bivalent antibody triggers clathrin-mediated endocytosis (CME), while FGFR1 tetramerization with multivalent antibody enhances internalization of the receptor by simultaneous engagement of CME and clathrin-independent (CIE), dynamin-dependent pathways [24, 25]. Thus, the oligomeric targeting proteins within the conjugates may, by controlling the arrangement of FGFR1 on the cell surface, upregulate internalization of the receptor-conjugate complex, improving drug delivery to the cancer cells. Importantly, the enhanced endocytosis was detected upon clustering of few other cell surface receptors, implicating that oligomerization of targeting molecules could serve as a universal approach for development of highly efficient drug carriers in conjugates targeting cancer biomarkers [26].

Here we present a modular system for generation of the oligomeric, high affinity, highly internalizing and effective cytotoxic conjugates targeting cancer-specific biomarkers. In our studies we employed FGFR1 as a model molecular target and engineered fibroblast growth factor 1 (FGF1) as a receptor-targeting molecule. Our data strongly indicate that oligomerization of targeting molecule constitutes an attractive strategy to improve efficiency of the conjugates targeting cancer-specific biomarkers.

## Methods

### Antibodies and reagents

The primary antibodies directed against FGFR1 (#9740), phospho-FGFR (pFGFR; #3476), ERK1/2 (#9102) and phospho-ERK1/2 (pERK1/2; #9101) were from Cell Signaling (Danvers, MA, USA). Anti-tubulin primary antibody (#T6557) was from Sigma-Aldrich (St Louis, MO, USA), and anti-FGF1 (sc-55520), anti-FGF2 (sc-365106) and anti-GST (sc-138) primary antibodies were from Santa Cruz Biotechnology (Dallas, TX, USA). Secondary antibodies coupled to HRP were from Jackson Immuno-Research Laboratories (Cambridge, UK).

### Cells

Human osteosarcoma cell line (U2OS) and human breast adenocarcinoma cell line (SKBR3) were obtained from American Type Culture Collection (ATCC) (Manassas, VA, USA). U2OS cells stably expressing FGFR1 (U2OS-R1) were obtained by transfection of U2OS cells with expression plasmid encoding FGFR1 [27]. Cells were cultured in 5% CO_2_ atmosphere at 37 °C in Dulbecco’s Modified Eagle’s Medium (Biowest, Nuaille, France) supplemented with 10% fetal bovine serum (FBS) (Thermo Fisher Scientific), antibiotics mix (100 U/mL penicillin and 100 μg/mL streptomycin) (Thermo Fisher Scientific), for U2OS-R1 additionally supplemented with 0.5 mg/mL geneticin (Thermo Fisher Scientific). Murine embryonic fibroblasts (NIH3T3) were from ATCC and were cultured in Dulbecco’s Modified Eagle’s Medium (Biowest) supplemented with 2% bovine serum and antibiotics mix (100 U/mL penicillin and 100 μg/mL streptomycin) (Thermo Fisher Scientific). Cells were grown in 5% CO_2_ atmosphere at 37 °C. Human lung cancer cell line DMS114 was obtained from ATCC and cultured in 5% CO_2_ atmosphere at 37 °C in Waymouth's medium (Thermo Fisher Scientific) supplemented with 10% FBS and antibiotics mix (100 U/mL penicillin and 100 μg/mL streptomycin) (Thermo Fisher Scientific). Cells were seeded onto tissue culture plates one day prior start of the experiments.

### Recombinant proteins

Fully glycosylated extracellular domain of FGFRs fused to the Fc fragment of human IgG1: FGFR1 IIIc (FGFR1-Fc) was produced as described previously by our group [28].

Based on the standard molecular biology methods, the AviTag sequence was introduced to the C-terminus of FGF1 and FGF1E [29], generating Avi-tagged proteins: FGF1-AviTag and FGF1E-AviTag. Both recombinant proteins were expressed in *E. coli* BL21(DE3) pLysS strain (Agilent Technologies, Santa Clara, CA, USA). Cells were grown at 37 °C until OD_600_ = 0.8, then protein expression was induced by addition of 1 mM IPTG, followed by incubation of cells at 30 °C for 16 h. Avi-tagged proteins were purified by heparin affinity chromatography on a HiTrap Heparin HP column (GE Healthcare, Chicago, IL, USA) eluted with 2 M NaCl, 20 mM Tris–HCl, 1 mM DTT, 0.1 mm PMSF and 1 mM EDTA. The identity and the purity of the proteins was confirmed by SDS-PAGE and western blotting.

Plasmids pET21a-Streptavidin-Alive (Addgene plasmid #20860) and pET21a-Streptavidin-Dead (Addgene plasmid #20859) were a kind gift from Alice Ting (Addgene, Watertown, MA, USA). SA-Alive and SA-Dead were expressed in *E. coli* BL21 CodonPlus(DE3)-RIL strain (Agilent Technologies) and purified using modified method described by Howarth et al. [30]. Briefly, cells were grown at 37 °C until OD_600_ = 0.8, then protein expression was induced by addition of 1 mM IPTG and cells were incubated at 30 °C for 16 h. Inclusion bodies containing SA variants were dissolved in buffer containing 50 mM Tris, 100 mM NaCl, 0.1 mg lysozyme, 0.5 mM PMSF pH 8.0 and sonicated (Vibra Cell, Sonics, Newtown, CT, USA) for 15 min, 60% amplitude, 5 s on/5 s off. Then sonicated inclusion bodies were centrifuged for 1 h, 4193 g, 4 °C. Next, pellet was resuspended in buffer containing 50 mM Tris, 100 mM NaCl, 1 mM EDTA, 10 mM DTT, 2% Triton X-100 pH 8.0 and incubated at 4 °C for 30 min constantly stirring. This procedure was repeated with buffer containing 50 mM Tris–HCl, 100 mM NaCl, 1 mM EDTA, 10 mM DTT pH 8.0. Next, purified inclusion bodies were dissolved in 6 M guanidinium hydrochloride, refolded by rapid dilution into stirring PBS buffer and incubated overnight at 4 °C. After incubation, the mixture was centrifuged for 15 min, 17,000×*g*, 4 °C to dispose of precipitates formed. Protein was then concentrated by addition of ammonium sulfate, as described by Howarth et al. [30] and centrifuged for 15 min, 17,000×*g*, 4 °C. Obtained streptavidin pellet was resuspended in a minimum volume of PBS and desalted by overnight dialysis. This method was sufficient to purify tetrameric tetravalent “Alive” streptavidin (SA-4A) and tetrameric biotin non-binding streptavidin “Dead” (SA-4D).

To generate chimeric streptavidin, inclusion bodies of SA-Alive and SA-Dead were dissolved in 6 M guanidinium hydrochloride, mixed in the desired ratio and refolded, as described above. Different SA tetramers were separated by affinity chromatography using a nickel–nitrilotriacetic acid column (Ni–NTA) (GE Healthcare) and eluted with imidazole gradient (0–300 mM) in 50 mM Tris–HCl. The elution buffer of purified proteins was exchanged to PBS using the Desalting HiTrap column (GE Healthcare). The identity and the purity of the proteins were confirmed by SDS-PAGE and western blotting.

Glutathione-*S*-transferase-BirA (GST-BirA) expression plasmid was a kind gift from Prof. Chris O’Callaghan from University of Oxford (Oxford, UK) [31]. GST-BirA was expressed in *E. coli* BL21 CodonPlus (DE3)-RIL strain (Agilent Technologies). Cells were grown at 37 °C until OD_600_ = 0.8, then protein expression was induced by addition of 1 mM IPTG and after that cells were incubated at 30 °C for 16 h. The clarified supernatant was applied to a GSTrap FF 5 mL column (GE Healthcare) equilibrated with 50 mM Tris–HCl, 150 mM NaCl, 0.025% Triton X-100, 1 mM PMSF pH 8.0. GST-BirA was eluted with 50 mM Tris–HCl, 150 mM NaCl, 15 mg/mL reduced glutathione pH 7.5. The identity and the purity of the proteins was confirmed by SDS-PAGE and western blotting.

The coding sequence of Affibody_HER2_ with an N-terminal KCKSGG and a C-terminal SGGSSGGSGGLPETGGHHHHHH motifs in pET11b was obtained from Gene Universal (Newark, DE) as a custom gene synthesis. The protein was expressed in *E. coli* Rosetta2(DE3)pLysS strain. Cells were grown in TB medium at 37ºC to OD_600_ = 1, then protein expression was induced by addition of 0.2 mM IPTG and cells were incubated at 25ºC ON. Cells were then harvested by centrifugation at 6000×*g* and the pellet was resuspended in 25 mM HEPES, 150 mM NaCl, 0.1% Triton X-100, 1 mM PMSF pH 8.0. Next, cells were homogenized by sonication and cell debris was separated by ultracentrifugation at 50,000×*g* at 4ºC for 1 h. The clarified cell lysate was loaded onto nickel–nitrilotriacetic acid (Ni–NTA) column (GE Healthcare) and eluted with an imidazole gradient (0–500 mM) in 25 mM HEPES, 154 mM NaCl, 5% glycerol pH 7.6. The elution buffer in purified proteins was exchanged to 25 mM HEPES, 154 mM NaCl, 5% glycerol pH 7.6 using the Desalting HiTrap column (GE Healthcare). FGF2_V_ and sortase A were produced as described previously by our group [32]. The identity and the purity of the proteins were confirmed by SDS-PAGE.

### Synthesis of GGG-PEG12-Biotin

As a first step, the H_2_N-GGG-PEG12-C-CONH_2_ peptide was synthesized by solid phase peptide synthesis (SPPS) method in the Fmoc strategy. The peptide was hydrolyzed from the resin with a mixture of TFA/EDT/TIS/H_2_O (% v/v/v, 95:2:2:1), triply precipitated in cold Et_2_O, purified by reverse-phase high-performance liquid chromatography (RP-HPLC), and lyophilized. Next, H_2_N-GGG-PEG12-C-CONH_2_ (50 mg, 43 μmol) and maleimide-biotin (58 mg, 130 μmol, 3 equiv.) were dissolved in 1000 μL of DMAc, and then DIPEA (38 μL, 215 μmol, 5 equiv.) was added. The reaction was carried out at 20 °C for 12 h. The solvent was then removed under vacuum, and GGG-PEG12-Biotin was purified by RP-HPLC and lyophilized. The identity of the product was confirmed by MALDI MS.

The reagents used for solid-phase peptide synthesis were as follows: amino Fmoc-Gly-OH, Fmoc-l-Cys(StBu)–OH, Fmoc-O2Oc–OH; COMU (1-[1-(cyano-2-ethoxy-2-oxoethylideneaminooxy)-dimethylamino-morpholino] uroniumhexafluorophosphate), EDT (ethane-1,2-dithiol), piperidine, TIS (triisopropylsilane), DIPEA (N,N-diisopropylethylamine), DMF (N,N-dimethylformamide), DCM (dichloromethane), TFA (trifluoroacetic acid) and were purchased from Iris Biotech GmbH (Marktredwitz, Germany). HPLC-pure acetonitrile and Et_2_O (diethyl ether) were obtained from Avantor (Gliwice, Poland). TentaGel S RAM resin (particle size: 90 μm, loading 0.21 mmol/g) was from Rapp Polymere GmbH (Tübingen, Germany). DMAc (N,N-dimethylacetamide) and maleimide-biotin (N-Biotinoyl-N'-(maleimidohexanoyl)hydrazine) were purhased from Merck (Darmstadt, Germany). Synergi 4 μm Fusion-RP 80 Å 250 × 10 mm^2^ LC column was from Phenomenex Inc.

### Biotinylation of Avi-tagged proteins

High-salt buffer in Avi-tagged protein sample was exchanged to reaction buffer containing 20 mM HEPES, 150 mM NaCl, 10 mM MgCl_2_ pH 7.0 using the Desalting HiTrap column (GE Healthcare). Then, 100 μM of protein was subjected to biotinylation by addition of 4 mM ATP, 2 μM GST-BirA, 150 μM D-biotin for 1 h at 30 °C. Next, the reaction mixture was supplemented with 2 mM ATP, 1 μM GST-BirA, 75 μM D-biotin and incubated for an additional 1 h at 30 °C. Biotinylated proteins were purified by affinity chromatography on a HiTrap Heparin HP column (GE Healthcare) and eluted with 2 M NaCl, 1 mM DTT, 0.1 mM PMSF and 1 mM EDTA. Successful attachment of biotin was demonstrated with SDS-PAGE, mass spectrometry and via interaction with SA.

### Biotinylation of proteins with sortase A

The conjugated proteins were diluted in 25 mM HEPES, 154 mM NaCl, 5 mM CaCl_2_, 2 mM TCEP pH 7.6 to final concentration of 35 uM, then G3PEG12-Biotin was added to a final concentration of 100 µM. Sortase A was then added to a final concentration of 2 μM and the mixture was incubated for 3 h for MMAE-FGF2V and 12 h for MMAE-Afiibody_HER2_ at 15 °C. Biotinylated MMAE-Affibody_HER2_ (MMAE-Affibody_HER2_-Biot) was purified by ion exchange chromatography using a SP Sepharose HiTrap column (GE Healthcare). Elution was performed in 25 mM HEPES, 0.5 M NaCl, 200 mM urea, 1 mM TCEP, CaCl_2_ 2 mM, pH 7.6. Biotinylated MMAE-FGF2_V_ (MMAE-FGF2_V_-Biot) was purified by affinity chromatography with a Heparin HiTrap column (GE Healthcare). Protein elution was performed using 25 mM HEPES, 2 M NaCl, 25 mM Na_2_SO_4_, 5% Glycerol pH 7.4.

### Mass spectrometry

Molecular mass of FGF1-AviTag-Biot was verified by MALDI-TOF MS (Applied Biosystems AB 4800+, Foster City, CA, USA) using α-cyano-4-hydroxycinnamic acid as a matrix.

### Assembling of various SA-based oligomers

Biotinylated FGF1E-AviTag-Biot was mixed with each SA variant in different ratios and incubated for 5–10 min at RT. The resulting protein mixtures were subjected to SDS-PAGE analysis. To purify FGF1-SA oligomers on a preparative scale, the protein components were mixed in the appropriate ratio (1:1) and incubated for 5–10 min at RT. The protein mixtures were subjected to size exclusion chromatography in PBS using a HiLoad 16/60 Superdex 200 prep grade column (GE Healthcare). This procedure was also used to obtain the tetrameric cytotoxic conjugate MMAE-FGF1E-AviTag-Biot-SA-4A. To obtain the MMAE-Affibody_HER2_-SA-4A complex, protein components were mixed in the appropriate ratio (1:1) and incubated for 5–10 min at RT. The protein mixtures were subjected to size exclusion chromatography in 25 mM HEPES, 0.25 mM NaCl, 5% glycerol pH 7.4 using Superdex 75 10/300 GL column (GE Healthcare), and then analyzed by SDS-PAGE with CBB staining. The MMAE-FGF2_V_-Biot was mixed with SA-4A in a 1:1 ratio and incubated for 5–10 min at RT. The resulting complex was subjected to SDS-PAGE with CBB staining and western blotting.

### Conjugation of proteins with MMAE

Conjugation of FGF1E-AviTag, FGF2_V_ and Affibody_HER2_ with monomethyl auristatin E (MMAE) was performed with protein concentration of 0.5 mg/mL dissolved in PBS with 1 mM EDTA pH 7.4. The protein was added to maleimidocaproyl-Val-Cit-PABC-monomethyl auristatin E (vcMMAE) (MedChem Express, Monmouth Junction) at five-fold molar excess of MMAE over protein cysteine residues and incubated for 2 h at RT. After incubation, MMAE-FGF1-AviTag was purified by ion exchange chromatography using the HiTrap SP Sepharose FF column (GE Healthcare). The resin was washed with washing buffer containing 25 mM HEPES, 5% glycerol pH 7.4 to remove unconjugated MMAE and then MMAE-FGF1-AviTag was eluted with elution buffer containing 25 mM HEPES, 0.5 M NaCl, 10 mM Na_2_SO_4_, 5% glycerol pH 7.4. The purity and the identity of conjugate were confirmed by SDS-PAGE.

### Circular dichroism measurements

CD spectra were recorded in the wavelength range of 195–260 nm in phosphate buffer (10 mM H_3_PO_4_, pH 7.4) at 25ºC on a Jasco J-715 spectropolarimeter (Jasco, Tokyo, Japan). Measurements were performed at protein concentration of 50 µM using a 0.2 mm cuvette with a slit width set to 2 nm and a response time of 1 s.

### BLI measurements

Binding analysis of recombinant proteins was performed using bio-layer interferometry (BLI) with ForteBio Octet K2 (Pall ForteBio, San Jose, CA, USA). To analyze interaction between FGF1-AviTag and FGFR1, the extracellular region FGFR1-Fc (10 μg/mL) was immobilized on ProtA biosensors (Pall ForteBio, San Jose, CA, USA). FGF1 protein was used as a control. Association and dissociation phases of the FGF1 (10 μg/mL) and FGF1-AviTag (10 μg/mL) were monitored in PBS buffer supplemented with 0.2% BSA and 0.05% Triton X-100.

To analyze binding ability between biotinylated proteins and streptavidin variants, 10 μg/mL of non-biotinylated proteins (FGF1-AviTag or FGF1E-AviTag or MMAE-FGF1E-AviTag) serving as controls and 10 μg/mL of biotinylated proteins (FGF1-AviTag-Biot or FGF1E-AviTag-Biot or MMAE-FGF1E-AviTag-Biot) were immobilized on AR2G biosensors (Pall ForteBio). Association and dissociation phases of the SA-4A (10 μg/mL) were monitored in PBS buffer supplemented with 0.2% BSA and 0.05% Triton X-100.

The binding ability of biotinylated MMAE-FGF2_V_-Biot or MMAE-Affibidy_HER2_-Biot to streptavidin was analyzed in comparison to non-biotinylated protein variants (MMAE-FGF2_V_ and MMAE-Affibody_HER2_) using SAX2 biosensors. Association and dissociation phases were monitored in PBS supplemented with 0.2% (w/v) BSA, 0.1% PEG 3.5 kDa, 0.05% (v/v) Triton X-100, and 10 mM (NH_4_)_2_SO_4_.

To determine the kinetic parameters of interaction of analyzed proteins with FGFR1, FGFR1-Fc was immobilized on ProtA biosensor (Pall ForteBio). Various concentrations of FGF1-SA oligomers: FGF1-AviTag-Biot-SA-4A (75–600 nM), FGF1-AviTag-Biot-SA-3A1D (75–600 nM), FGF1-AviTag-Biot-SA-2A2D (75–600 nM), FGF1-AviTag-Biot-SA-1A3D (75–600 nM) or MMAE-FGF1E-AviTag-Biot-SA-4A (75–600 nM) were applied on the sensors and association, and dissociation were measured in PBS with 0.2% BSA and 0.05% Triton X-100. Kinetic constants (k_on_, k_off_, and K_D_) were calculated using global fitting with the 2:1 “heterogeneous ligand” mode with ForteBio Data Analysis 11.0 software (Pall ForteBio, San Jose, CA, USA).

### Activation of FGFR1 and downstream signaling cascades

To analyze the effect of recombinant proteins on FGFR1 activation and initiation of downstream signaling cascades, serum-starved NIH3T3 cells were incubated for 15 min at 37 °C with FGF1 (0.2, 2, 5, 10, 50 ng/mL), as a control, or FGF1-AviTag (0.2, 2, 5, 10, 50 ng/mL) in the presence of heparin (10 U/mL). Cells were lysed in Laemmli buffer and subjected to SDS-PAGE and western blotting. The experiments were performed analogously for FGF1E-AviTag-Biot (2, 5, 20, 100 ng/mL) and FGF1-SA oligomers: FGF1-AviTag-Biot-SA-4A (50 ng/mL), FGF1-AviTag-Biot-SA-3A1D (50 ng/mL), FGF1-AviTag-Biot-SA-2A2D (50 ng/mL), FGF1-AviTag-Biot-SA-1A3D (50 ng/mL) with adequately higher concentrations of FGF1, as control to provide the cells with equal molar concentrations of targeting molecule.

### Flow cytometry

FGF1E-AviTag was labeled with Alexa Fluor 488 C_5_ maleimide (Thermo Fisher Scientific) according to manufacturer’s protocol and then biotinylated. U2OS-R1 cells were seeded onto 12-well plates (200,000 cells per well) in full medium and left to attach overnight. Then, medium was removed, cells were washed with PBS buffer and starved with serum-free medium for 4 h. Next, plates were cooled on ice, and labeled FGF1E-AviTag-Biot (500 ng/mL) or labeled FGF1E-AviTag-Biot (500 ng/mL) assembled with non-labeled SA-4A (500 ng/mL) were added to the cells in the presence of heparin, in a serum-free medium supplemented with 1% BSA. After 40 min of incubation on ice, the cells were moved to 37 °C for 15 min to allow for internalization. Then, the medium was removed and the cells were washed with serum-free medium supplemented with 0.2% BSA pH 3.5 (three times, 5 min) and then with PBS buffer (three times, 1 min). Cells were subsequently detached with 10 mM EDTA in PBS buffer, pH 8.0, harvested by centrifugation and resuspended in PBS supplemented with 1% BSA. Cells were analyzed using a NovoCyte 2060R Flow Cytometer and NovoExpress software (ACEA Biosciences, San Diego, CA).

### Confocal microscopy

As for flow cytometry FGF1E-AviTag was labeled with Alexa Fluor 488 C_5_ maleimide (Thermo Fisher Scientific) and then biotinylated. U2OS-R1 cells were seeded at a density of 8000 cells per well in 96-well plates (Greiner Bio-One, Kremsmunster, Austria, #655096) in complete medium and left to attach overnight. Then, medium was removed, cells were washed with PBS buffer and starved in serum-free medium for 4 h. Next, the plates were cooled on ice for 20 min, and labeled FGF1E-AviTag-Biot (500 ng/mL) alone or labeled FGF1E-AviTag-Biot (500 ng/mL) assembled with non-labeled SA-1A3D (500 ng/mL), SA-2A2D (500 ng/mL), SA-3A1D (500 ng/mL) or SA-4A (500 ng/mL) were added to cells in the presence of heparin and DAPI in serum-free medium. After 40 min of incubation on ice, cells were transferred to Opera Phenix confocal microscope (Perkin Elmer, Waltham, MA, USA) preheated to 37 °C and with 5% CO_2_ atmosphere. Cells were imaged every 5 min for 60 min to monitor internalization. Harmony software (version 4.9; Perkin Elmer) was used for image acquisition and analysis. At least eight 16-bit images with 2048 × 2048 pixels resolution were acquired for each experimental condition using a 40/1.1 water immersion objective. The integral intensity of the labeled FGF1E-AviTag-Biot spots was calculated and expressed in arbitrary units (AU). Cell number was determined by detection of nuclei using the DAPI signal. All data were normalized to cell number. Images were assembled in Photoshop (Adobe) with linear adjustments of contrast and brightness only.

### Cytotoxicity assay

The cytotoxicity of the MMAE-FGF1E-AviTag-Biot and MMAE-FGF1E-AviTag-Biot-SA-4A were tested on FGFR1-negative cell line (U2OS) and FGFR1-positive cell lines (U2OS-R1 and DMS114). Cells were plated at 5000 cells per well in 96-well plates and incubated for 24 h at 37 °C in the presence of 5% CO_2_. Serial dilutions of FGF1E-AviTag-Biot and FGF1E-AviTag-Biot-SA-4A (from 0.01 to 100 nM), as controls, and MMAE-FGF1E-AviTag-Biot, MMAE-FGF1E-AviTag-Biot-SA-4A (from 0.01 to 100 nM) were incubated with the cells for 96 h in the presence of heparin (10 U/mL). Monomeric forms of recombinant FGF1 were added in four-fold higher concentrations to provide the cells with equal amounts of FGF1 targeting molecule and MMAE payload. Cell viability was measured using PrestoBlue™ Cell Viability Reagent (Thermo Fisher Scientific), according to the manufacturer's protocol. Fluorescence emission at 590 nm (excitation at 560 nm), reflecting the viability of the cells, was measured using Infinite M1000 PRO plate reader (Tecan, Männedorf, Switzerland). Every experiment was conducted in three independent repeats. Statistical analyses were done using t-test. EC_50_ values were calculated based on the Hill equation using Origin 7 software (Northampton, MA).

The cytotoxicity of MMAE-FGF2_V_-Biot and MMAE-FGF2_V_-Biot-SA-4A were tested on FGFR1-positive cell lines (U2OS-R1), and MMAE-Affibody_HER2_-Biot-SA-4A was tested on HER2-positive cell line (SKBR3). Cells were plated at 5000 cells per well in 96-well plates and incubated for 24 h at 37 °C in the presence of 5% CO_2_. U2OS-R1 cells were incubated for 96 h with MMAE-FGF2_V_-Biot (from 0.01 to 100 nM), SA-4A (from 0.01 to 100 nM) or a mixture of MMAE-FGF2_V_-Biot and SA-4A (each from 0.01 to 100 nM) in the presence of heparin (10 U/mL). Serial dilutions of MMAE-Affibody_HER2_ or MMAE-Affibody_HER2_-Biot-SA-4A (from 0.01 to 100 nM) were incubated with SKBR3 cells for 96 h. The monomeric form of MMAE-Affibody_HER2_ was added at four-fold higher concentrations to provide the cells with equal amounts of targeting molecule and MMAE payload. Cell viability was measured using PrestoBlue™ Cell Viability Reagent (Thermo Fisher Scientific), according to the manufacturer's protocol. Fluorescence emission at 590 nm (excitation at 560 nm), reflecting cell viability, was measured using an Infinite M1000 PRO plate reader (Tecan, Männedorf, Switzerland). Each experiment was performed in triplicate.

## Results

### Preparation of streptavidin scaffold for controlled oligomerization of FGF1

**Fig. 1:**
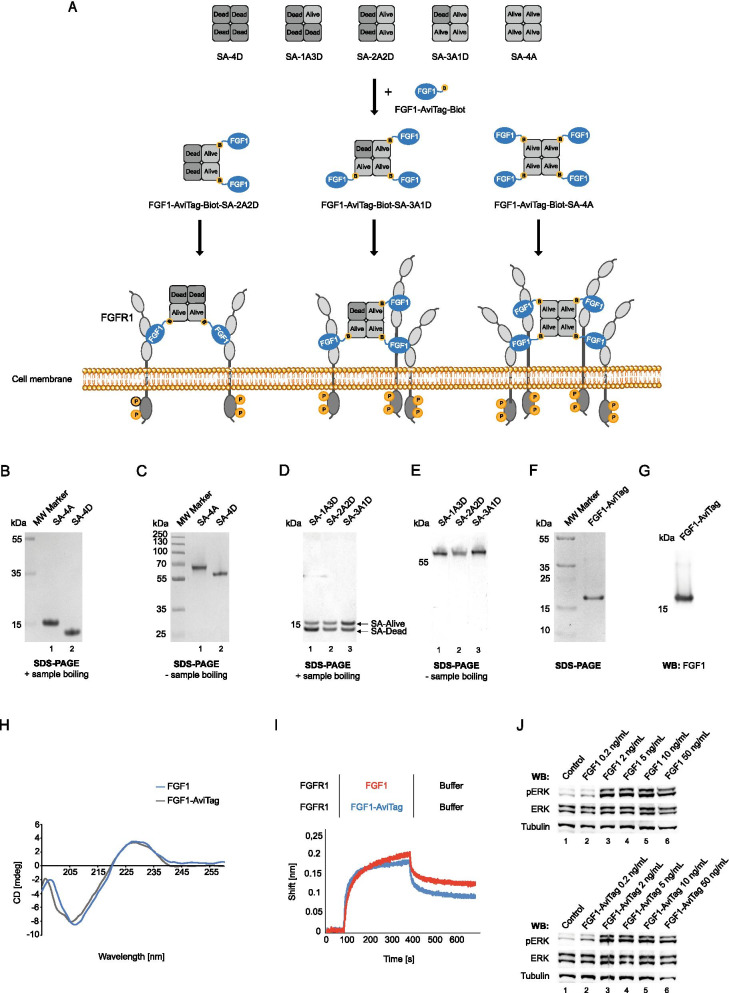
Basic building blocks for development of SA-FGF1 oligomers. **A** Strategy for development of FGF1-SA oligomers. In this approach SA tetramers containing from 0 to 4 binding sites for biotinylated ligands are obtained by mixing wild type SA-Alive-HisTag and non-biotin-binding SA-Dead mutant. FGF1-AviTag will be enzymatically biotinylated by GST-BirA and then assembled with various SA scaffolds, leading to formation of FGF1-SA oligomers with distinct potential for clustering of FGFR1. **B** and **C** Separately purified SA-Alive-HisTag and non-biotin-binding SA-Dead. The purity and identity of streptavidin variants were confirmed by SDS-PAGE (CBB staining), presence of the HisTag on the SA-Alive version allows for comparison of SA variants composition. To maintain the tetrameric form of the protein, SA samples in C were not subjected to thermal denaturation. **D** and **E** Variants of streptavidin tetramers with varying valency. Separately purified SA-Alive-HisTag and SA-Dead were mixed, yielding all possible combinations. Due to the presence of the His-Tag on SA-Alive version (ensuring affinity to metal ions), metal-affinity chromatography was applied to separate various combinations of SA tetramers. The purity and identity of obtained SA variant were confirmed by SDS-PAGE. Upon boiling, samples were separated into monomers (**D**). The ratio of two SA-bands demonstrates protein tetramers containing from 0 to 4 binding sites for biotinylated ligands. To preserve the tetrameric form of SA, samples in E were not subjected to thermal denaturation. **F** and **G** FGF1-AviTag protein was purified by heparin affinity chromatography. Purified protein was analyzed by SDS–PAGE under reducing condition (**F**) and western blotting (WB) with antibody directed against FGF1 (**G**). **H** CD spectra of wild type FGF1 and FGF1-AviTag confirming preservation of 2D structure of FGF1 upon incorporation of AviTag. **I** BLI comparison of FGF1 and FGF1-AviTag interaction with FGFR1. The extracellular region of FGFR1 was immobilized on BLI sensors and incubated either with FGF1 and FGF1-AviTag. The association and dissociation profiles were measured. **J** FGF1-AviTag is able to activate FGFR1. Serum-starved NIH3T3 cells were incubated for 15 min with different concentrations of FGF1 (positive control) or FGF1-AviTag in the presence of heparin. Cells were lysed and activation of FGFR1 assessed with western blotting using antibodies recognizing phosphorylated key tyrosine within intracellular FGFR tyrosine kinase domain (pFGFR) and receptor-downstream ERK (detected with antibodies recognizing phosphorylated ERK (pERK). The level of tubulin served as a loading control

We and others have shown that clustering of cell surface receptors boosts their endocytosis, typically by simultaneous engagement of multiple endocytic pathways [16, 25, 26, 33]. Here, we decided to build on these findings and to develop a modular, easily exchangeable system for generation of oligomeric, highly internalizing cytotoxic conjugates (Fig. 1A). As a molecular target we selected FGFR1, a receptor overproduced by numerous cancer types. As oligomerization hub we employed engineered variants of streptavidin (SA) developed by Howarth et al. [30]. Wild type SA is a highly stable tetrameric protein capable of simultaneous binding of four biotinylated ligands [30]. His-Tag was introduced to wild type SA, resulting in SA “Alive” (A) [30]. The mutant “Dead” (D) variant of SA still assembles into a tetramer, but is unable to interact with biotinylated ligands [30]. Combined refolding of purified A and D variants followed by affinity purification allows for development of mixed SA with a different number of biotin binding sites: none (SA-4D), one (SA-1A3D), two (SA-2A2D), three (SA-3A1D) and four (SA-4A) [30] (Fig. 1A). SA tetramers of different valency can be used for controlled oligomerization of biotinylated receptor ligand (Fig. 1A). As a targeting molecule we employed an engineered variant of natural FGFR1 ligand, fibroblast growth factor 1 (FGF1) fused C-terminally with AviTag sequence (FGF1-AviTag), which allows for BirA-mediated, site specific biotinylation and assembly of multivalent complexes with different SA tetramers (Fig. 1A).

Initially, we prepared individual components required to generate distinct FGF1-SA oligomers. SA is very efficiently produced in *E. coli* and results in protein found in inclusion bodies [30]. Inclusion bodies containing SA-A and SA-D were purified separately and subjected to refolding by rapid dilution to yield SA tetramers. As shown in Fig. 1B, highly pure SA-4A and SA-4D variants were obtained (SA tetramers disassemble into monomers upon sample boiling in SDS-PAGE). SA-A variant migrates more slowly on SDS-PAGE gels due to incorporation of His-Tag (Fig. 1B). SA tetramers are very stable and their oligomeric state is retained even in the presence of SDS (when sample was not boiled) [30]. As expected, purified SA-A and SA-D assembled into tetramers, confirming the correct quaternary structure of SA after refolding (Fig. 1C).

Next, separately purified SA-A and SA-D were mixed and subjected to refolding followed by affinity purification using Ni–NTA column. The higher number of SA-A subunits within SA tetramer the higher affinity for Ni–NTA was observed, which was due to the presence of His-Tag on SA-A. As shown in Fig. 1D, monovalent SA-1A3D (lane 1; seen on SDS-PAGE as two bands upon sample boiling—underrepresented SA-A and overrepresented SA-D, confirming 1:3 ratio), bivalent SA-2A2D (lane 2; SA-A and SA-D bands of equal intensity confirming 2:2 composition of tetramer) and trivalent SA-3A1D (lane 3; seen on SDS-PAGE as two bands upon sample boiling—overrepresented SA-A and underrepresented SA-D, confirming 3:1 ratio) were obtained. The preservation of SA quaternary structure in mixed SA tetramers was confirmed with SDS-PAGE without sample boiling (Fig. 1E). In agreement, the higher the valency of SA tetramer, the slower migration (due to presence of His-Tag on SA-A subunit) was observed (Fig. 1E).

### Development of modified FGF1 for self-assembly with SA

Next, we modified the FGFR1 ligand, FGF1, to enable its assembly with SA variants. To this end, we genetically fused FGF1 with AviTag, produced FGF1-AviTag in *E. coli* and purified to homogeneity using affinity chromatography (Fig. 1F). The identity of FGF1-AviTag was confirmed with western blotting using antibodies specific for FGF1 (Fig. 1G). To study whether AviTag incorporation altered the secondary structure of FGF1 we employed circular dichroism (CD). As shown in Fig. 1H, the CD spectra of wild type FGF1 and FGF1-AviTag were virtually indiscernible. Furthermore, we employed biolayer interferometry (BLI) to assess the impact of AviTag on the interaction of FGF1 with FGFR1. We immobilized the recombinant extracellular region of FGFR1 fused to the Fc fragment (FGFR1ecd.Fc) on BLI sensors and incubated the receptor with wild type FGF1 and FGF1-AviTag. The binding curves for both studied proteins were almost identical (Fig. 1I). Finally, using NIH3T3 fibroblasts we showed that FGF1-AviTag activated FGFR1 and downstream-signaling kinases ERK1/2 as efficiently as wild type FGFR1 (Fig. 1J). All these data univocally demonstrate that FGF1-AviTag is fully functional and therefore suitable for application as a targeting molecule in SA-based FGF1 oligomers.

### Assembly of SA-FGF1 complexes of varying valency

**Fig. 2:**
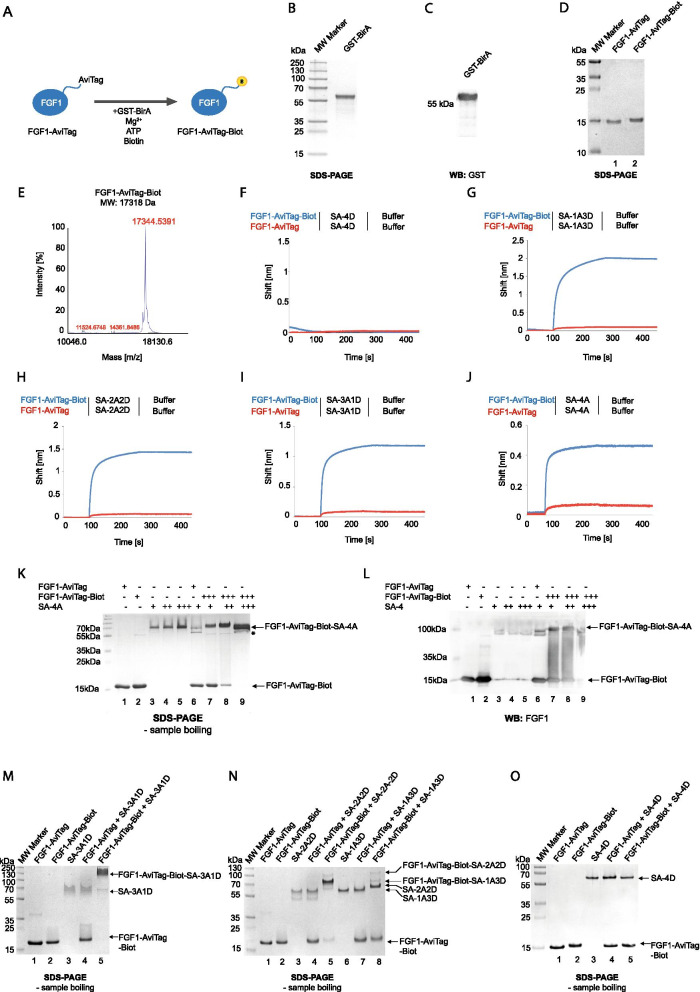
Assembly of SA-FGF1 oligomers. **A** BirA-mediated site-specific FGF1-AviTag biotinylation. FGF1 was fused to the AviTag acceptor peptide and this Avi-tagged protein was selectively biotinylated by GST-BirA enzyme, leading to the formation of site-specific mono-biotinylated ligand. Attached biotin is marked in yellow. **B** and **C** GST-BirA was purified by glutathione affinity chromatography. Protein purity was analyzed by SDS–PAGE (CBB staining) (**B**) and western blotting (WB) with anti-GST antibody (**C**). **D** Biotinylation of FGF1-AviTag. The efficiency of the biotinylation (seen as upshift in protein migration) and purity of FGF1-AviTag-Biot was analyzed by SDS-PAGE (CBB staining) **E** Mass spectrometry analysis of purified FGF1-AviTag-Biot. The molecular mass of FGF1-AviTag-biot was assessed by MALDI MS. The theoretical MW of the proteinaceous core of FGF1-AviTag is 17092 Da, and 17,318 Da after biotinylation. **F–J** BLI comparison of FGF1-AviTag and FGF1-AviTag-Biot interaction with various SA variants. FGF1-AviTag and FGF1-AviTag-Biot were chemically immobilized on BLI sensors and incubated with SA-4D (**F**), SA-1A3D (**G**), SA-2A2D (**H**), SA-3A1D (I), SA-4D (**J**). The association and dissociation profiles were measured. **K** and **L** Assembling of FGF1-AT-Biot-SA-4A oligomer. FGF1E-AviTag and biotinylated variant of this protein were mixed with SA-4A in various ratios and incubated for 5–10 min at RT. Then, proteins mixes were subjected to SDS-PAGE analysis and CBB staining (**K**) and western blotting (**L**); *most likely self-assembly form of FGF1-AviTag or partial SA dissociation product upon SDS treatment. **M–O** Assembling of various FGF1-SA oligomers. Protein components were mixed in 1:1 ratio and incubated for 5–10 min at RT. Then, protein mixes were subjected to SDS-PAGE analysis and CBB staining

In the next step we subjected FGF1-AviTag to the site-specific enzymatic biotinylation with biotin ligase BirA that incorporates biotin to a lysine residue within the AviTag (Fig. 2A) [34]. We produced recombinant GST-tagged BirA (GST-BirA) and confirmed its purity and identity with SDS-PAGE (Fig. 2B) and western blotting (Fig. 2C). Next, we subjected FGF1-AviTag to BirA-mediated biotinylation. The incorporation of biotin to FGF1-AviTag decreased mobility of FGF1-AviTag on SDS-PAGE gels in relation to an unmodified protein (Fig. 2D). Importantly, the efficiency of GST-BirA-mediated biotinylation of FGF1-AviTag was very high, as virtually no unmodified FGF1-AviTag was detected (Fig. 2D). Additionally, we confirmed the successful biotinylation of FGF1-AviTag with mass spectrometry (Fig. 2E).

We employed BLI to study the interaction of biotinylated FGF1-AviTag (FGF1-AviTag-Biot) with purified SA variants. FGF1-AviTag and FGF1-AviTag-Biot were immobilized on BLI sensors and incubated with SA-4D (Fig. 2F), SA-1A3D (Fig. 2G), SA-2A2D (Fig. 2H), SA-3A1D (Fig. 2I) and SA-4A (Fig. 2J). As expected, we observed no interaction between FGF1-AviTag-Biot and SA-4D (Fig. 2F). In contrast, formation of complexes between FGF1-AviTag-Biot and all SA variants containing at least one A subunit were readily detected (Fig. 2G–J). Importantly, BLI data confirmed assembly of complexes with the desired architecture as a result of the biotin-SA interaction, as no binding between non-biotinylated FGF1-AviTag and SA was detected (Fig. 2G–J). The recorded binding curves were characterized by fast association rates and virtually no dissociation, which is in agreement with an extremely high affinity of SA for biotin and suggests the formation of very stable complexes between SA variants and FGF1-AviTag-Biot.

Since FGF1-AviTag-Biot-SA interaction was very strong, we wondered if FGF1-AviTag-Biot complexes could be visualized on SDS-PAGE gels. As shown in Fig. 2K, incubation of FGF1-AviTag-Biot with increasing concentrations of SA-4A resulted in efficient assembly of FGF1-AviTag-Biot-SA-4A complexes, as no unreacted FGF1-AviTag-Biot was detected at highest SA-4A concentration used (Fig. 2K, lanes 7–9). Furthermore, in western blotting experiments with anti-FGF1 antibodies FGF1-AviTag-Biot was detected in high molecular weight complexes upon incubation with SA-4A (Fig. 2L, lanes 7–9). Similar results were obtained for FGF1-AviTag-Biot and SA-3A1D (Fig. 2M, lane 5), SA-2A2D (Fig. 2N, lane 5) and SA-1A3D (Fig. 2N, lane 8). No complexes between FGF1-AviTag-Biot and SA-4D were detected, as expected (Fig. 2O, lane 5). All these data suggest successful assembly of highly stable streptavidin-based FGF1 architectures with the desired oligomeric state.

### Isolation of SA-FGF1 complexes of different valency with improved binding to FGFR1

**Fig. 3:**
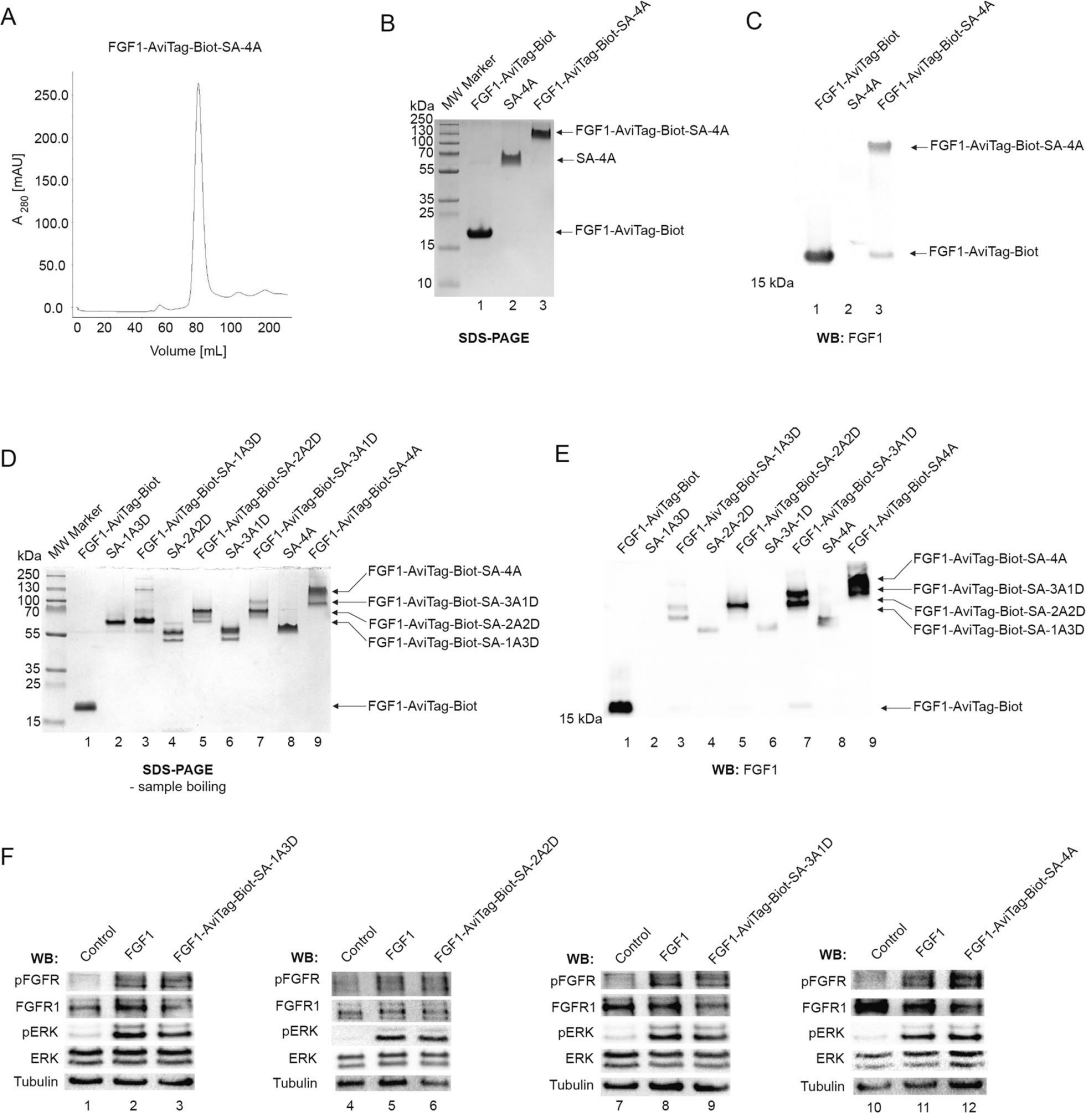
Purification of functional SA-FGF1 oligomers. **A** Size exclusion chromatography of FGF1-AviTag-Biot-SA-4A oligomer. The absorbance spectra were monitored at 280 nm. **B** and **C** Analysis of purified FGF1-AviTag-Biot-SA-4A complex. To analyze the efficiency of the folding and purity of the complex, SDS-PAGE (**B**) and western blotting with antibodies recognizing FGF1 (**C**) were performed. To maintain the tetrameric form of the protein, prepared samples were not subjected to thermal denaturation. **D** and **E** Analysis of various purified FGF1-SA oligomers (sequentially from monomer to tetramer). The purified complexes were subjected to SDS-PAGE analysis (**D**) and western blotting with antibodies recognizing FGF1 (**E**). Both methods excluded thermal denaturation of samples to maintain the tetrameric form of proteins. **F.** FGF1-SA oligomers are able to activate FGFR1. Serum-starved NIH3T3 cells were incubated for 15 min with FGF1-AviTag-Biot-SA-1A3D (50 ng/mL), FGF1-AviTag-Biot-SA-2A2D (50 ng/mL), FGF1-AviTag-Biot-SA-3A1D (50 ng/mL) or FGF1-AviTag-Biot-SA-4A (50 ng/mL) and adequately higher concentrations of FGF1, as a control, to provide the cells with equal amounts of FGF1 targeting molecule. Proteins were added in the presence of heparin. Cells were lysed and activation of FGFR1, and receptor-downstream signaling (using antibodies recognizing phosphorylated key tyrosine within intracellular FGFR tyrosine kinase domain (pFGFR) and receptor-downstream ERK (detected with antibodies recognizing phosphorylated ERK (pERK) signaling was assessed with western blotting. The level of tubulin served as a loading control

Based on optimized conditions for complex assembly between SA variants and FGF1-AviTag-Biot, we proceeded to isolation of preparative amounts of particular FGF1-AviTag-Biot-SA oligomers. For this purpose, the assembled complexes were subjected to gel filtration. The reaction mixture of SA-4A and FGF1-AviTag-Biot was eluted from the gel filtration column mostly as a single fraction, indicating efficient assembly of the SA-4A-based FGF1 tetramer (Fig. 3A). Similar data were obtained for other SA-based complexes (data not shown). Using SDS-PAGE and western blotting, we confirmed high purity of isolated FGF1 oligomer (Fig. 3B, lane 3) and presence of FGF1 in high molecular weight complexes composed of SA (Fig. 3C, lane 3). Similarly, we purified milligram quantities of all other FGF1-SA complexes, as demonstrated in Fig. 3D and E.

Next, we studied whether FGF1 in SA-based oligomers retained ability to bind cell-surface FGFR1. Serum-starved NIH3T3 fibroblasts were incubated with wild type monomeric FGF1 or with SA-FGF1 oligomers and activation of FGFR1 and receptor-dependent kinases ERK1/2 was assessed with western blotting. As shown in Fig. 3F, all FGF1-SA oligomers induced phosphorylation of FGFR1 and ERK1/2 to the same extent as wild type FGF1.

**Fig. 4:**
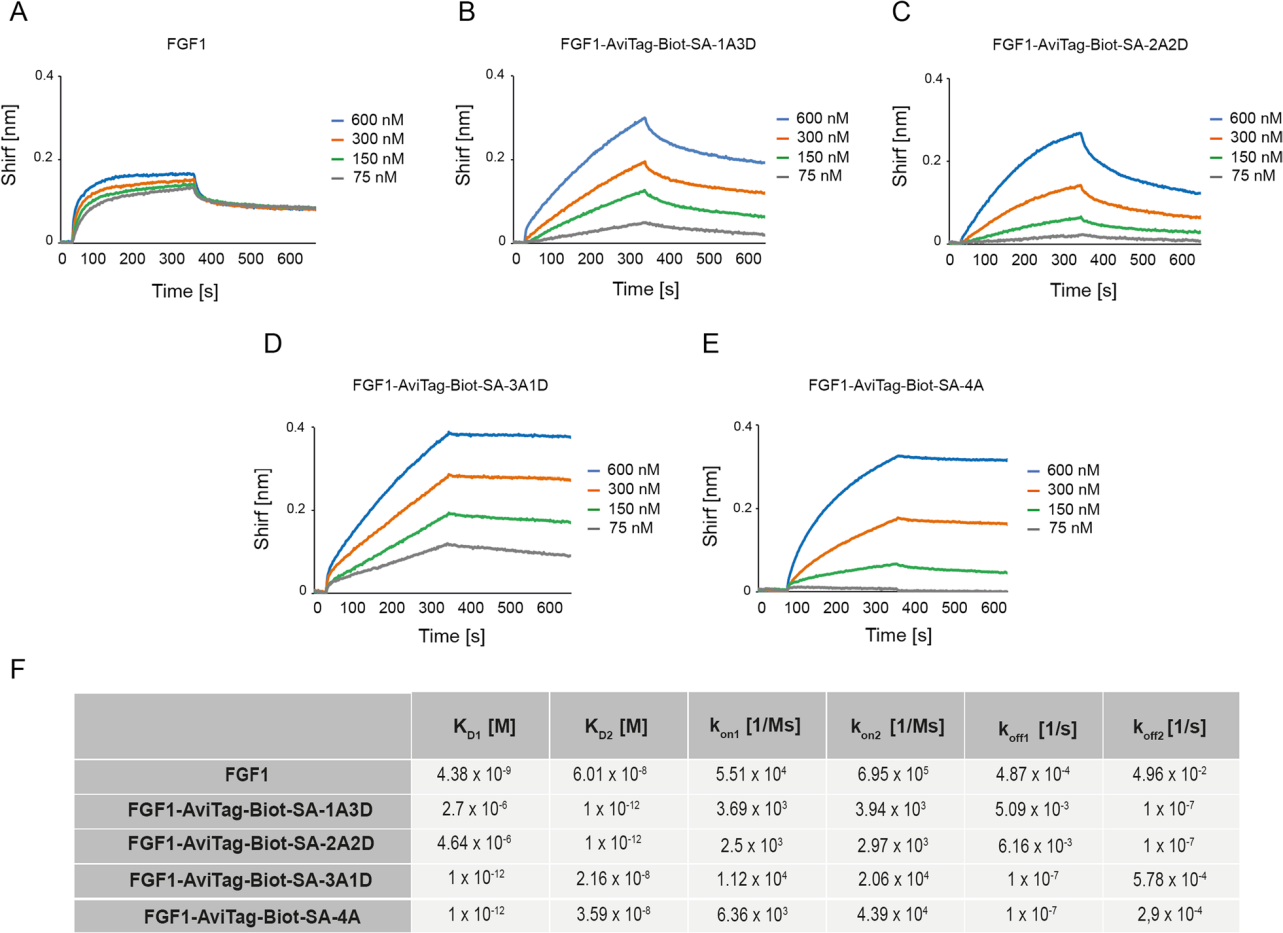
Kinetic parameters of FGF1-SA oligomers interaction with FGFR1. BLI-determined kinetic parameters of the interaction between FGF1 (**A**), FGF1-AviTag-Biot-SA-1A3D (**B**), FGF1-AviTag-Biot-SA-2A2D (**C**), FGF1-AviTag-Biot-SA-3A1D (**D**) or FGF1-AviTag-Biot-SA-4A (**E**) and FGFR1, respectively. The extracellular region of FGFR1 was immobilized on BLI sensors and incubated with various concentrations of FGF1-SA oligomers. K_D_, k_on_, and k_off_ were calculated using global fitting with the 2:1 “heterogeneous ligand” with ForteBio Data Analysis 11.0 software (**F**)

To study the impact of FGF1 oligomerization on the kinetics of growth factor interaction with FGFR1 we employed BLI. To this end, we immobilized FGFR1ecd.Fc on BLI sensors and incubated the receptor with different concentrations of wild type FGF1 and FGF1-SA oligomers. We assessed k_on_, k_off_ and K_D_ for all studied proteins. As shown in Fig. 4, all FGF1-SA oligomers efficiently interacted with FGFR1. We observed that monomeric and dimeric FGF1-SA complexes displayed similar affinity for FGFR1 as the wild type protein (Fig. 4A–C, F), while trimeric and tetrameric variants showed largely enhanced binding to the receptor (Fig. 4D–F). The improved FGFR1 binding by trimeric and tetrameric FGF1-SA oligomers was largely due to decreased dissociation rates (k_off_) and suggests formation of the highly stable, subnanomolar FGF1-SA oligomer–FGFR1 complex.

### Engineering of tetravalent cytotoxic conjugate targeting FGFR1

**Fig. 5:**
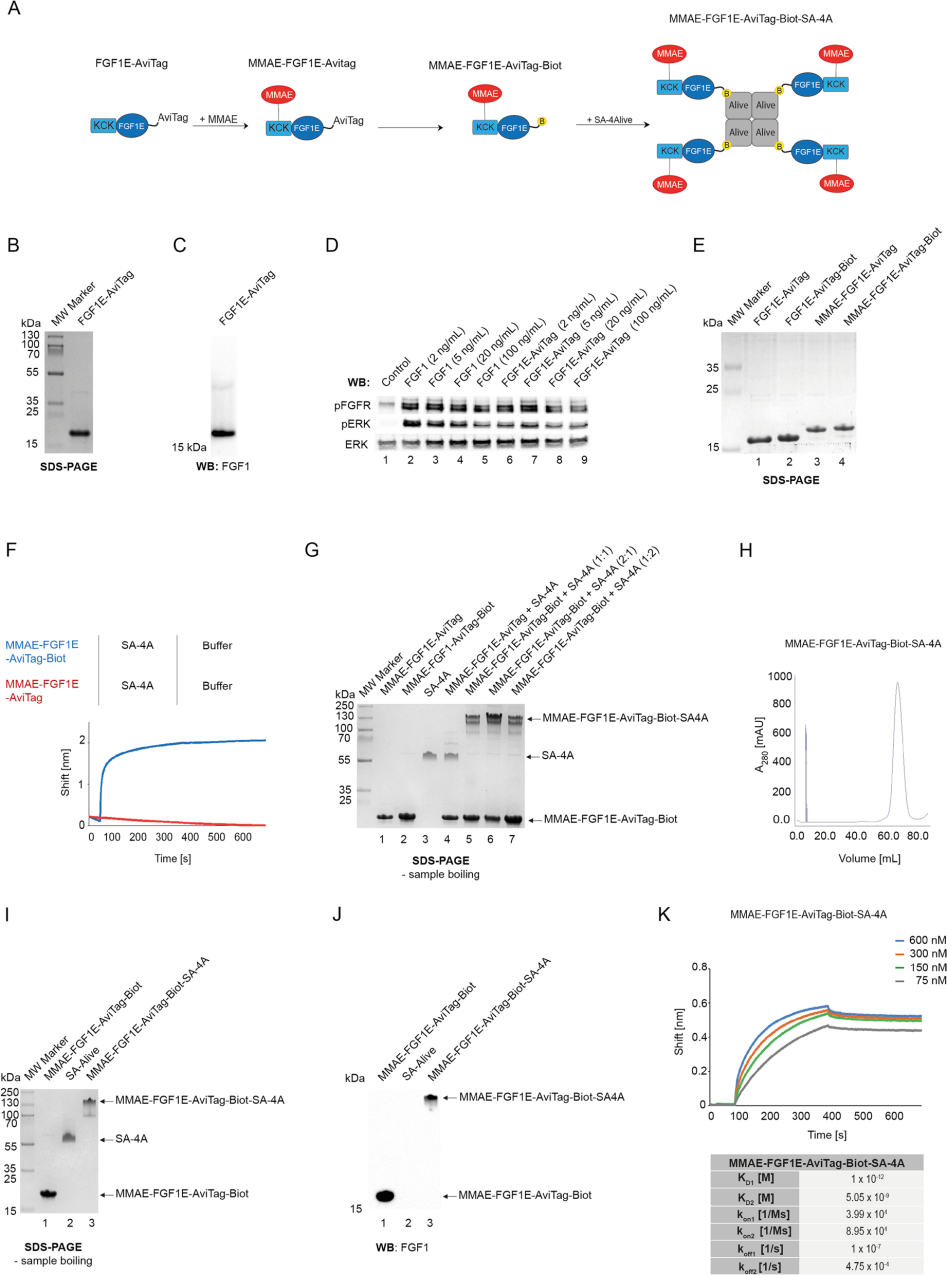
Development of tetrameric FGF1-SA. **A** Strategy for generation of the cytotoxic FGF1-SA oligomer. FGF1E-AviTag was conjugated to the cytotoxic compound MMAE via N-terminal cysteine flanked by two lysines. The conjugated protein was then biotinylated and assembled with tetrameric SA-4A to yield a cytotoxic tetrameric conjugate. Conjugated N-terminal cysteine is marked in blue, attached cytotoxic molecules are marked in red and attached biotin is marked in yellow. **B** and **C** FGF1E-AviTag protein was purified by heparin affinity chromatography. Using SDS-PAGE (**B**) and western blotting (**C**) with antibodies directed against FGF1, the purity and identity of protein were verified. **D** FGF1E-AviTag-Biot is able to activate FGFR1. Serum-starved NIH3T3 cells were incubated for 15 min with FGF1 (positive control) or with different concentrations of FGF1E-AviTag-Biot with the presence of heparin. Cells were lysed and activation of FGFR1 (pFGFR), and receptor-downstream signaling (pERK) was assessed with western blotting. The signal of non-modified FGFR and ERK served as a loading control. **E** Conjugation of FGF1E-AviTag with cytotoxic MMAE. The efficiency of the conjugation and biotinylation and purity of obtained MMAE-FGF1E-AviTag-Biot were confirmed by SDS-PAGE and CBB staining. **F** BLI comparison of MMAE-FGF1E-AviTag and MMAE-FGF1E-AviTag-Biot interaction with SA-4A. Both conjugates were chemically immobilized on BLI sensors and incubated with SA-4A. The association and dissociation profiles were measured. **G** Assembling of MMAE-FGF1E-AviTag-Biot-SA-4A oligomer. MMAE-FGF1E-AviTag and MMAE-FGF1E-AviTag-Biot were mixed with SA-4A in various ratios and incubated for 5–10 min at RT. Then, proteins mixes were subjected to SDS-PAGE analysis. **H** Size exclusion chromatography of MMAE-FGF1E-AviTag-Biot-SA-4A oligomer. The absorbance spectra were monitored at 280 nm. **I.** and **J.** The efficiency of the folding and purity of MMAE-FGF1E-AviTag-Biot-SA-4A were analyzed with SDS-PAGE (**I**) and western blotting with antibodies recognizing FGF1 (**J**). To maintain the tetrameric form of the protein, samples were not subjected to thermal denaturation. **K** BLI-determined kinetic parameters of the interaction between MMAE-FGF1E-AviTag-Biot-SA-4A and FGFR1. The extracellular region of FGFR1 was immobilized on BLI sensors and incubated with various concentrations of MMAE-FGF1E-AviTag-Biot-SA-4A. K_D_, k_on_, and k_off_ were calculated using global fitting with the 2:1 “heterogeneous ligand” with ForteBio Data Analysis 11.0 software

Based on the largely improved affinity for FGFR1 and the simplicity of preparation we selected the tetrameric variant of FGF1-SA as a basis for development of selective cytotoxic conjugates targeting cancer cells overproducing FGFR1. To further improve the stability of the FGF1-SA tetramer we used the mutant variant of FGF1, FGF1E, with three mutations stabilizing structure of the protein (Q40P, S47I, H93G), three native cysteines exchanged to serine residues (C16S, C83S, C117S) to eliminate non-specific attachment of the cytotoxic drug and an N-terminal KCKSGG sequence (abbreviated as KCK) providing a highly reactive cysteine for efficient and site-specific attachment of the cytotoxic payload (Fig. 5A) [29]. In the strategy for preparation of SA-based tetrameric conjugate, we designed FGF1E-AviTag, a mutant of FGF1E with the C-terminal AviTag for a site-specific biotinylation of protein (Fig. 5A). Maleimide-thiol chemistry allows for selective conjugation of cysteine residue within the KCKSGG linker with monomethylauristatin E (MMAE), a potent microtubule polymerization inhibitor and a cytotoxic drug (Fig. 5A). Subsequently, BirA-mediated, site-specific biotinylation of MMAE-FGF1E-AviTag allows for the assembly of a tetrameric FGF1-SA cytotoxic conjugate specific against FGFR1 (Fig. 5A).

We cloned and produced a highly pure recombinant FGF1E-AviTag, as verified using SDS-PAGE (Fig. 5B) and western blotting (Fig. 5C). To demonstrate that the introduced mutations and tags did not affect FGF1 ability to bind FGFR1, we performed signaling studies using NIH3T3 fibroblasts. MMAE-FGF1E-AviTag activated FGFR1 and ERK1/2 to the same extent as the wild type FGF1 (Fig. 5D). We further verified that FGF1E-AviTag was efficiently biotinylated (Fig. 5E, lane 2) and subsequently conjugated with MMAE (Fig. 5E, lane 3 and 4), which was visible as alterations in migration on SDS-PAGE gels. We confirmed the biotin-dependence of the interaction between biotinylated MMAE-FGF1E-AviTag and streptavidin using BLI (Fig. 5F) and SDS-PAGE (Fig. 5G), resulting in a tetrameric cytotoxic conjugate, named MMAE-FGF1E-AviTag-Biot-SA-4A. Next, we employed gel filtration to isolate milligram amounts of MMAE-FGF1E-AviTag-Biot-SA-4A. As shown in Fig. 5H, MMAE-FGF1E-AviTag-Biot-SA-4A was eluted mostly as a single peak from the gel filtration column. SDS-PAGE (Fig. 5I, lane 3) and western blotting (Fig. 5J, lane 3) confirmed high purity of the successfully assembled MMAE-FGF1E-AviTag-Biot-SA-4A conjugate.

Since tetrameric FGF1-AviTag-Biot-SA-4A displayed a significantly enhanced affinity for FGFR1, we used BLI to analyze if the tetrameric conjugate MMAE-FGF1E-AviTag-Biot-SA-4A conjugate retained improved binding to FGFR1. MMAE-FGF1E-AviTag-Biot-SA-4A was characterized by subnanomolar affinity for FGFR1, a value much higher compared to monomeric wild type FGF1 and highly similar to unconjugated FGF1 tetramer (Fig. 5K).

### Enhanced cellular uptake of the tetrameric conjugate targeting FGFR1

**Fig. 6:**
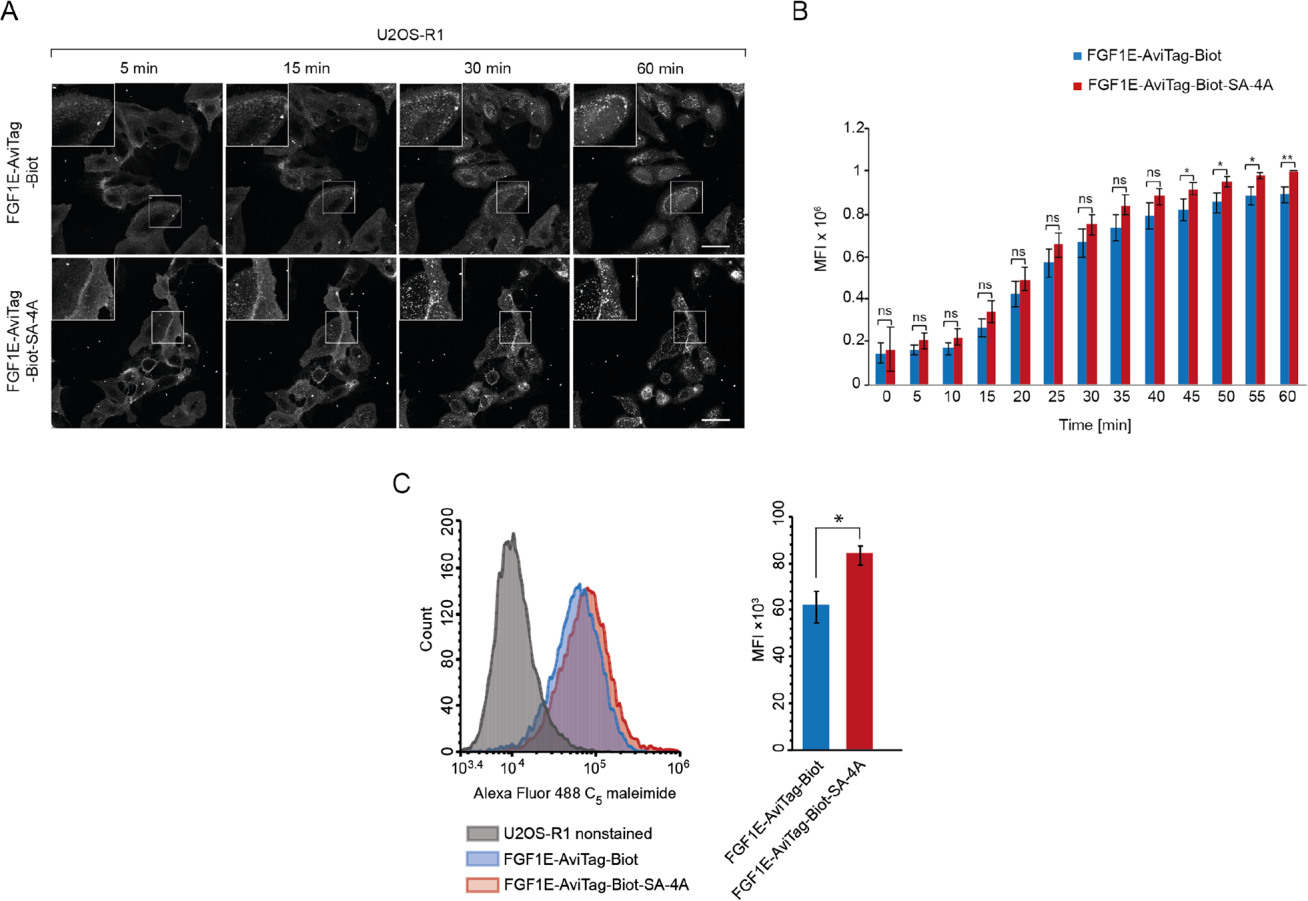
Enhanced internalization of tetrameric FGF1-SA conjugates via FGFR1-mediated endocytosis. **A** Live cell imaging of the kinetics of FGF1E-AviTag-Biot and FGF1E-AviTag-Biot-SA-4A endocytosis. U2OS-R1 cells were incubated on ice for 40 min with Alexa Fluor 488 C_5_ maleimide-labeled FGF1E-AviTag-Biot in the presence or absence of SA-4A, shifted to 37 °C and imaged live for 60 min using spinning disk confocal microscopy. Images taken at the indicated time points are shown. Scale bar represents 50 μm. **B** Quantitative analysis of endocytosis of FGF1E-AviTag-Biot and FGF1E-AviTag-Biot-SA-4A. Average values from five independent live cell imaging experiments ± SEM are shown. t-test was used to assess statistical significance; *p < 0.05, **p < 0.01, ***p < 0.0001, n.s.- not significant. **C** Efficiency of FGF1E-AviTag-Biot and FGF1E-AviTag-Biot-SA-4A internalization analyzed by flow cytometry. Serum-starved U2OS-R1 cells were treated with Alexa Fluor 488 C_5_ maleimide FGF1E-AviTag-Biot and FGF1E-AviTag-Biot mixed with SA-4A. After 40 min incubation on ice, cells were transferred to 37 °C for 30 min, and then subsequently analyzed by NovoCyte 2060R Flow Cytometer. Average values of three independent experiments ± SD are shown. t-test was used to assess statistical significance (n = 3) * p < 0.05

We have previously demonstrated that the high affinity of ligands promotes their internalization via FGFR1-mediated endocytosis [25, 35]. Therefore, we analyzed if enhanced receptor binding of MMAE-FGF1E-AviTag-Biot-SA-4A is accompanied by increased efficiency of its uptake via FGFR1-mediated endocytosis. We fluorescently labelled biotinylated FGF1E-AviTag at the cysteine residue within the KCK linker and incubated labelled protein with U2OS-R1 cells stably producing FGFR1 in the presence, or absence of SA-4A. We employed live cell quantitative confocal microscopy to assess differences in endocytosis of monomeric FGF1E-AviTag-Biot and tetrameric FGF1E-AviTag-Biot-SA-4A. The microscopical analysis of the kinetics of monomeric FGF1E-AviTag-Biot and tetrameric FGF1E-AviTag-Biot-SA-4A endocytosis revealed enhanced uptake of the tetramer in comparison to the monomeric counterpart (Fig. 6A). Quantitative analyses confirmed higher internalization of the tetramer (Fig. 6B). Enhanced internalization was not detected for dimeric and trimeric FGF1E-AviTag-Biot variants (Additional file 1: Fig. S1). Additionally, we employed flow cytometry for quantitative measurements of cellular uptake of monomeric FGF1E-AviTag-Biot and tetrameric FGF1E-AviTag-Biot-SA-4A. In agreement with confocal microscopy, flow cytometry experiments revealed increased internalization of the tetrameric variant (Fig. 6C).

All these data demonstrate successful development of tetrameric conjugate, MMAE-FGF1E-AviTag-Biot-SA-4A, with improved FGFR1 binding characteristics and enhanced internalization via FGFR1-dependent endocytosis.

### Superior cytotoxicity of oligomeric MMAE-FGF1E-SA-4A

We wondered whether enhanced FGFR1 binding and elevated cellular uptake of the MMAE-FGF1E-AviTag-Biot-SA-4A conjugate would ultimately result in improved cytotoxicity against FGFR1-overproducing cells. Since we observed that the longer the kinetics of the cellular uptake of conjugates was measured, the greater differences between the monomer and the tetramer were detected, we expected that these differences will be even more pronounced at later time points, where the cytotoxicity of the conjugates is assessed.

**Fig. 7:**
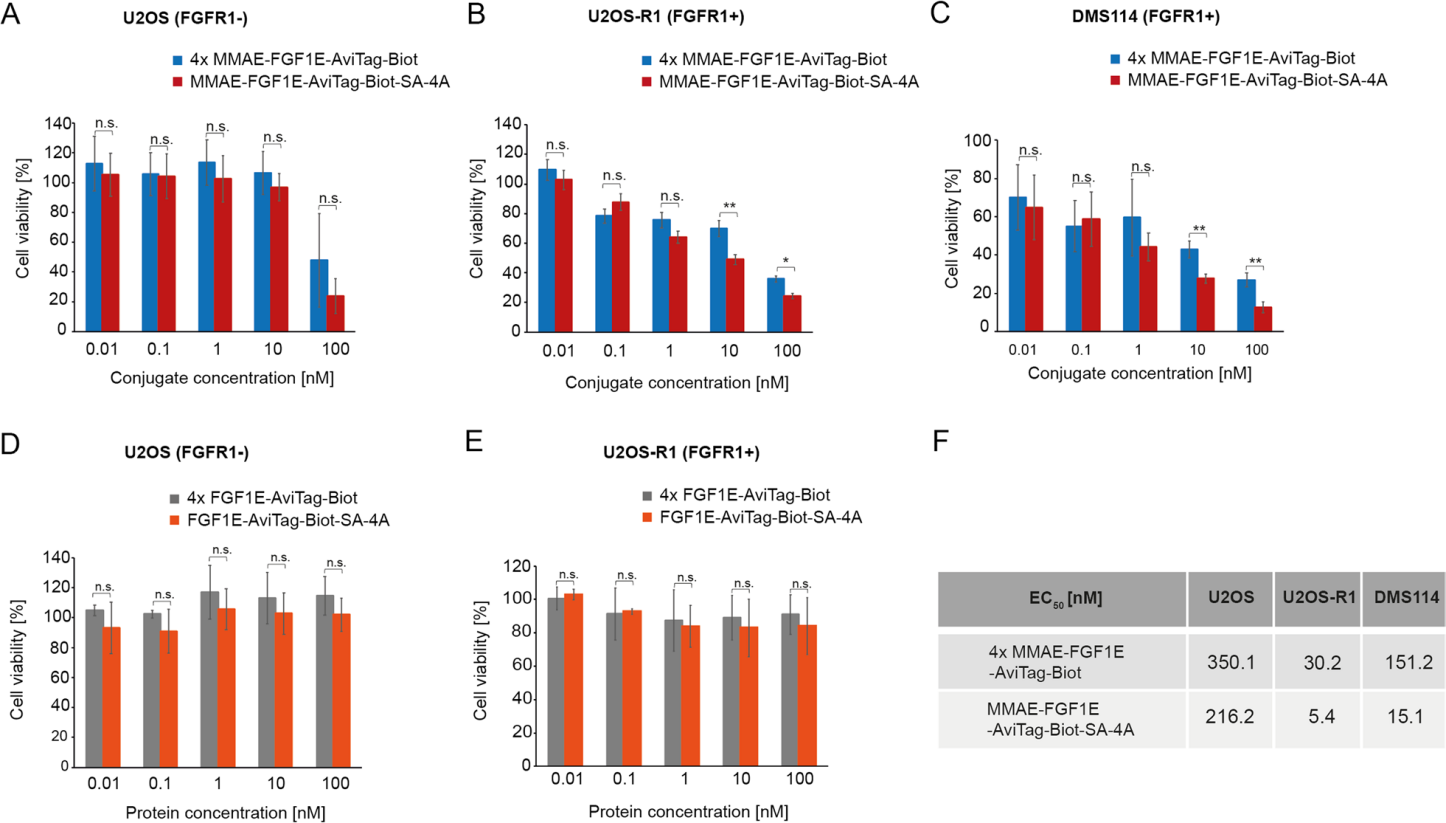
Superior cytotoxicity of the tetrameric FGF1-SA conjugate against FGFR1 producing cells. **A–C** Cytotoxic potential of MMAE-FGF1E-AviTag-Biot and MMAE-FGF1E-AviTag-Biot-SA-4A was measured in various cell lines: U2OS (**A**), U2OS-R1 (**B**) and DMS114 (**C**). **D** and **E** Control cytotoxicity of non-conjugated FGF1E-AviTag-Biot and FGF1E-AviTag-Biot-SA-4A was measured in U2OS (**D**) and U2OS-R1 (**E**) cells. All cells were treated with the indicated agents at various concentrations for 96 h and their viability was assessed with the Presto Blue assay (**A**–**E**). Results are mean values from three independent experiments ± SD. Statistical significance: *p < 0.05, **p < 0.01, ***p < 0.0001, n.s.- not significant. **F** EC_50_ values of analyzed proteins were calculated based on the Hill equation using Origin 7 software (Northampton, MA). 4 ×—monomeric non-conjugated FGF1E-AviTag-Biot and monomeric conjugated MMAE-FGF1E-AviTag-Biot were used in fourfold higher concentrations in the experiments to provide cells with equal molar concentrations of drug and targeting molecule

We used U2OS-R1 as a model cells stably producing FGFR1 and small cell lung cancer cells DMS114 characterized by high level of FGFR1 expression. USOS cells lacking detectable FGFR1 were used as control. We treated the cells with various concentrations of MMAE-FGF1E-AviTag-Biot-SA-4A conjugate or with four-fold higher concentrations of monomeric MMAE-FGF1E-AviTag-Biot to provide the cells with equal amounts of FGF1 targeting molecule and MMAE payload. For U2OS control cells we observed cytotoxic effect only at the highest tested concentration of conjugates and there was no significant difference between the monomeric and tetrameric conjugates (Fig. 7A). We observed concentration-dependent high cytotoxicity of the conjugates for both FGFR1-positive cell lines tested (Fig. 7B and C). Importantly, the tetrameric conjugate MMAE-FGF1E-AviTag-Biot-SA-4A was significantly more efficient in inducing cell death compared to its monomeric counterpart (Fig. 7B and C). In agreement, the measured EC_50_ values revealed that the tetrameric MMAE-FGF1E-AviTag-Biot-SA-4A conjugate is from six to tenfold more potent than monomeric MMAE-FGF1E-AviTag-Biot (Fig. 7F). Additionally, we demonstrated that unconjugated proteins display no cytotoxicity to cells, regardless of the absence (Fig. 7D) or presence (Fig. 7E) of FGFR1 on their surface.

Since FGF1 efficiently binds to several FGFRs, we tested the applicability of our strategy by using more specific FGF2 as an FGFR targeting molecule, which preferentially recognizes FGFR1 and FGFR3 (isoforms c) [36]. We used FGF2_v_ variant with cysteines 78 and 96 mutated to serines, additionally containing the N-terminal KCKSGG linker for efficient drug conjugation and the C-terminal LPETGG sequence allowing for a site-specific biotinylation via sortase A-mediated ligation. FGF2_v_ was efficiently conjugated to MMAE, biotinylated and combined with SA-4A to yield the tetravalent conjugate MMAE-FGF2_v_-Biot-SA-4A (Additional file 1: Fig. S2A-C) [32]. Importantly, oligomerization of MMAE-FGF2_v_-Biot-SA with SA-4A resulted in increased cytotoxicity of the tetrameric conjugate in relation to the monomeric variant (Additional file 1: Fig. S2D).

To validate our approach for another cancer relevant marker receptor, HER2, we employed Affibody_HER2_, a highly stable, high affinity engineered three helix bundle specifically recognizing HER2 [37]. We modified Affibody_HER2_ to ensure site-specific attachment of MMAE and biotin. Affibody_HER2_ was efficiently conjugated to MMAE, biotinylated in a site-specific manner and assembled into tetramers with SA-4A (Additional file 1: Fig. S3A and B). Next, SKBR3, a human breast cancer cell line expressing HER2, was used to investigate the cytotoxicity of monomeric MMAE-Affibody_HER2_ an tetrameric MMAE-Affibody_HER2_-Biot-SA-4A. As shown in Additional file 1: Fig. S3C, the tetrameric variant displayed enhanced cytotoxicity in comparison to its monomeric counterpart.

All these data demonstrate that enhanced FGFR1 binding and elevated FGFR1-dependent endocytosis of the oligomeric MMAE-FGF1E-AviTag-Biot-SA-4A conjugate result in its enhanced cytotoxicity against FGFR1-overproducing cells in relation to the monomeric conjugate. Furthermore, our data implicate that oligomerization of targeting molecules serves as a general approach to improve the efficiency of cytotoxic conjugates.

## Discussion

Conventional chemotherapy is currently the most frequently used cancer treatment approach, but, although being successful to some extent, it has many drawbacks including high-dose requirement, poor pharmacokinetic properties, unspecific drug targeting and numerous side effects [38]. Targeted anti-cancer therapeutics aim to overcome these limitations by selective and precise delivery of toxic agents into tumors and at the same time omitting healthy cells [9, 39]. Targeted anti-cancer approaches largely rely on the presence of specific macromolecules on the surface of tumor cells, which are preferably not expressed, or are produced at very low levels by normal cells [5, 7]. To date, numerous cancer-specific cell-surface marker proteins have been identified and growth factor receptors constitute one group of tumor markers with elevated expression in different cancer types [15, 40]. Different drug targeting agents, including monoclonal antibodies, antibody fragments and receptor ligands that precisely recognize growth factor receptors, have been covalently conjugated to potent cytotoxic drugs and successfully used to selectively destroy cancer cells [2, 41, 42]. The effectiveness of targeted anti-cancer approach with the use of cytotoxic conjugates depends on the selective recognition of the tumor marker by the targeting molecule within the conjugate and its ability to deliver the cytotoxic payload to the tumor cell interior, typically via endocytosis of marker-conjugate complex. Thus, novel strategies to improve tumor markers recognition and the cellular uptake of conjugates are highly desirable [43, 44].

FGFR1 is a cell surface receptor overexpressed by several tumors, including breast, lung, ovarian, head and neck and bladder cancers and constitutes an attractive tumor marker for development of targeted therapeutics [44]. Low molecular weight chemical inhibitors, ligand traps, antibodies and their fragments, and a few cytotoxic conjugates have been developed to either block abnormal FGFR1-dependent signaling that facilitates cancer cell proliferation and survival, or to selectively kill FGFR1-overproducing cells [16, 18–20, 45]. Recently, we have shown that the spatial distribution of FGFR1 in the plasma membrane determines efficiency and mechanism of FGFR1 endocytosis. [24, 25]. These findings prompted us to design a modular, self-assembling, easily exchangeable system for development of multivalent, high affinity and highly internalizing FGFR1-targeting molecules. As FGFR1-targeting part for the whole oligomeric construct we used engineered variant of FGF1. For the controlled oligomerization of FGF1 we employed modified streptavidin, which had already been proven successful as an oligomerization scaffold [33]. Using this system, we assembled complexes with the desired architecture and obtained highly pure FGFR1-targeting molecules of different oligomeric states, from monomer to tetramer. While all FGF1 oligomers were capable of FGFR1 binding and activation, trimeric and tetrameric FGF1 displayed largely increased affinity for FGFR1 in comparison to the monomeric ligand. We have previously observed a similar phenomenon for tetravalent anti-FGFR1 antibody and coiled-coil-triggered FGF1 oligomers and in both cases the elevated affinity for FGFR1 was due to considerably decreased dissociation rates [25, 46]. Importantly, improved FGFR1 binding was accompanied by enhanced cellular uptake of oligomeric FGF1, indicating that SA-FGF1 multivalent ligands, by affecting the spatial distribution of FGFR1, control its cellular trafficking and may constitute effective targeting molecules for selective drug delivery.

Based on all the promising characteristics of tetravalent FGF1-SA, we decided to evaluate its potential as a drug vehicle in cytotoxic conjugates targeting FGFR1-oveproducing cells. Tetravalent conjugate MMAE-FGF1E-AviTag-Biot-SA-4A is up to tenfold more cytotoxic than its monomeric MMAE-FGF1E-AviTag-Biot counterpart, independently of cell line tested. These data suggest that improved receptor binding and enhanced cellular uptake observed upon controlled tetramerization of FGF1 with SA are reflected in largely upgraded cytotoxicity of the FGF1-based conjugate. Based on our findings we postulate that oligomerization of the targeting agents by streptavidin or other scaffolds constitutes an attractive strategy to improve selective drug delivery in anti-cancer therapies employing cytotoxic conjugates. Up to date, several conjugates targeting FGFR1 have been developed and our data implicate that their potency may be further improved by their oligomerization [16]. In our studies, we selected FGF1 as a “proof of principle” high affinity FGFR1 ligand. However, it is important to notice that FGF1 interacts with all FGFRs and this broad specificity may be disadvantageous in the clinic in targeting a particular tumor type [47]. Therefore, we also prepared tetrameric conjugates based on more specific FGFR ligand, FGF2, and demonstrated its superiority over the monomeric FGF2-based conjugate. Further improvement in the selectivity of FGFR targeting with oligomeric conjugates could be achieved by using FGFs from FGF7 subfamily that are highly specific towards FGFR2b and FGFR1b [47]. Alternatively, antibody fragments highly selective against individual FGFRs could be employed instead of FGFs [24, 35, 48, 49].

The optimal size of the cytotoxic conjugate is still a matter of debate, as on the one hand two small molecules will be rapidly removed from the bloodstream, whereas overly large conjugates will be limited in tumor penetration [50]. The tetrameric conjugate based on FGF1 and streptavidin described in this study is about 130 kDa, thus its size is highly similar to monoclonal antibodies commonly used in the ADC approach [50]. However, if for some combinations of targeting molecule and oligomerization scaffold the resulting molecular weight of the conjugate is too high, there are several opportunities for the optimization, like employing peptides as receptor targeting agents or using smaller oligomerization agents, like coiled coil motifs.

The strategy for development of oligomeric cytotoxic conjugates presented herein is not limited to FGFR1. It was demonstrated that cell surface crosslinking of transferrin receptor TfnR, ErbB receptor family members (HER2 and EGFR) or acetylcholine receptor enhances their uptake [26]. Consequently, here we demonstrated that tetramerization of a conjugate constructed based on HER2 specific Affibody_HER2_ improved its cytotoxicity. TfnR receptor clustering alters also intracellular trafficking of the receptor, promoting its lysosomal delivery instead of recycling [33]. Clustering-mediated enhanced lysosomal targeting may be beneficial for anti-cancer therapies with cytotoxic conjugates, as it prevents recycling-mediated removal of the conjugates from cancer cells. Therefore, oligomerization of targeting molecules in cytotoxic conjugates emerges as an attractive tool to elevate specificity and efficiency of drug delivery, leading to increased potency of the conjugates.

The SA-based system for preparation of oligomeric cytotoxic conjugates described in this study can be easily adapted to other cancer markers as well. By engineering the ligands towards site-specific drug conjugation and biotinylation followed by self-assembly with SA tetramers, oligomeric conjugates of selected specificity can be easily and rapidly developed. In numerous tumors different cell surface proteins are overexpressed and their simultaneous targeting with multi-specific oligomers could enhance efficiency of the therapy and overcome cancer drug resistance [51–55]. Furthermore, some cancer biomarkers, such as HER2, are well known for their poor internalization, which limits their targeting with cytotoxic conjugate [56, 57]. Multi-specific conjugates build based on the SA system can bring HER2 and other highly internalizing receptor close together, enhancing the uptake of HER2 targeting conjugates.

It is well described that cancer cells develop resistance to chemotherapy [58, 59] One of emerging solutions to overcome this therapy limitation is the simultaneous application of several drugs with distinct mode of action. Our group has recently demonstrated beneficial effect of dual-warhead conjugates against FGFR1-positive cancer cells [60]. The modularity of the SA-based system may facilitate the rapid development of dual- or multi-warhead conjugates, in which different drugs are linked to targeting molecules and SA within the oligomer. Furthermore, SA-based oligomeric cytotoxic conjugates could easily incorporate additional functional moieties: fluorophores for conjugate imaging or modifications enhancing lysosomal delivery [61].

Summarizing, our data confirm the applicability of the FGF1-SA oligomers as highly effective drug delivery vehicles for the selective treatment of FGFR1-producing cancer cells. Furthermore, we conclude that multivalent targeting molecules, due to their high affinity for receptors and superior cellular trafficking, may constitute attractive alternatives to conventional drug delivery vehicles, like antibodies. Importantly, the SA-based model presented in this study constitutes a modular system that can be adapted to other cancer markers and further functionalized to increase the efficacy of targeted therapy in the future.

## Conclusions

- Self-assembly, modular system for development of oligomeric cytotoxic conjugates against FGFR1-overproducing cancer cells has been developed,
- Oligomeric cytotoxic conjugates are characterized by well-defined architecture, site-specific attachment of the cytotoxic drug and improved affinity for the cancer-specific cell surface receptor,
- Oligomeric cytotoxic conjugate displays enhanced internalization into cancer cells via receptor-mediated endocytosis, which is reflected by their significantly elevated cytotoxicity,
- Due to its modularity, presented approach can be easily adapted for generation of highly effective, self-assembly oligomeric cytotoxic conjugates against other cancer-specific markers.

## Supplementary Information

**Additional file 1: Fig. S1.** Internalization of various FGF1-SA oligomers via FGFR1-mediated endocytosis.A. Live cell imaging of endocytosis of monomeric FGF1E-AviTag-Biot and FGF1E-AviTag-Biot in combination with different SA variants. U2OS-R1 cells were incubated on ice for 40 min with Alexa Fluor 488 C5 maleimide-labeled FGF1E-AviTag-Biot alone or in the presence of SA variants of different valency (from 1 to 3). Then, cells were transferred to 37°C and imaged live for 60 min using a spinning disk confocal microscope. Images taken at the indicated time points are shown. The scale bar represents 50 m. B. Quantitative analysis of endocytosis of FGF1E-AviTag-Biot alone or in combination with different variants of streptavidin. Mean values from three live cell imaging experiments +/-SEM are shown. **Fig. S2.** Development of the tetrameric MMAE-FGF2v-Biot-SA-4AA - B. FGF2V was conjugated to the cytotoxic compound MMAE via N-terminal cysteine flanked by two lysines. Then, the conjugated protein was biotinylated using sortase A and assembled with tetrameric SA-4A to yield a cytotoxic tetrameric conjugate. The efficiency of conjugation, biotinylation and correctness of complex assembly were confirmed by SDS-PAGE with CBB staining (A) and western blotting with antibodies directed against FGF2 (B). Thermal denaturation of the SDS-PAGE samples was skipped to preserve the tetrameric form of proteins. C. Biotinylation of the cytotoxic conjugate was confirmed by BLI by measuring the interaction of MMAE-FGF2V and MMAE-FGF2V-Biot with streptavidin-bearing SAX2 biosensors. Association and dissociation profiles were measured. D. The cytotoxic potential of the tetrameric conjugate MMAE-FGF2v-Biot-SA-4A was evaluated in U2OS-R1 cell line. Cells were treated with MMAE-FGF2V-Biot in the presence or absence of SA-4A at various concentrations for 96 h. Then, cells viability was assessed with the Presto Blue assay. Results are mean values from three experiments +/-SEM. **Fig. S3.** Development of the tetrameric MMAE-AffibodyHER2-Biot-SA-4A targeting HER2 receptor.A. AffibodyHER2 was conjugated with cytotoxic MMAE via an N-terminal KCK motif. Then, conjugated protein was biotinylated with using sortase A and assembled with SA-4A to obtain a tetrameric MMAE-AffibodyHER2-Biot-SA-4A conjugate. The purity and identity of the proteins at each reaction step were verified by SDS-PAGE with CBB staining. To preserve the tetrameric form of the protein during SDS-PAGE, the thermal denaturation step was omitted. B. BLI comparison of MMAE-AffibodyHER2 and MMAE-AffibodyHER2-Biot binding to streptavidin using SAX2 biosensors. Association and dissociation profiles were measured. C. The cytotoxic potential of monomeric MMAE-AffibodyHER2 and tetrameric MMAE-AffibodyHER2-Biot-SA-4A was measured in the SKBR3 cell line. Cells were treated with MMAE-AffibodyHER2 or MMAE-AffibodyHER2-Biot-SA-4A at various concentrations for 96 h. Then, cell viability was assessed with the Presto Blue assay. Results are mean values from three experiments +/-SEM. 4x – monomeric MMAE-AffibodyHER2 was used at four times higher concentrations in the experiments to provide cells with equal molar concentrations of drug and targeting molecule.

## Data Availability

The datasets used in this study are available from the corresponding author on reasonable request.

## Abbreviations

BLI: Biolayer interferometry
CIE: Clathrin independent endocytosis
CME: Clathrin mediated endocytosis
FGFs: Fibroblast growth factors
FGFR1: Fibroblast growth factor receptor 1
GST: Glutathione S-transferase
HLA: Human leukocyte antigen
MHC: Major histocompatibility complex
MMAE: Monomethyl auristatin E
SA: Streptavidin
RTK: Receptor tyrosine kinase
