# Supplementary material for: Modular self-assembly system for development of oligomeric, highly internalizing and potent cytotoxic conjugates targeting fibroblast growth factor receptors
Source: J Biomed Sci. 2021 Oct 11;28:69. doi: 10.1186/s12929-021-00767-x (PMC8504119; doi:10.1186/s12929-021-00767-x)
Supplement: Supplementary file 1 — Additional file 1: Fig. S1. Internalization of various FGF1-SA oligomers via FGFR1-mediated endocytosis.A. Live cell imaging of endocytosis of monomeric FGF1E-AviTag-Biot and FGF1E-AviTag-Biot in combination with different SA variants. U2OS-R1 cells were incubated on ice for 40 min with Alexa Fluor 488 C5 maleimide-labeled FGF1E-AviTag-Biot alone or in the presence of SA variants of different valency (from 1 to 3). Then, cells were transferred to 37°C and imaged live for 60 min using a spinning disk confocal microscope. Images taken at the indicated time points are shown. The scale bar represents 50 m. B. Quantitative analysis of endocytosis of FGF1E-AviTag-Biot alone or in combination with different variants of streptavidin. Mean values from three live cell imaging experiments +/-SEM are shown. Fig. S2. Development of the tetrameric MMAE-FGF2v-Biot-SA-4AA - B. FGF2V was conjugated to the cytotoxic compound MMAE via N-terminal cysteine flanked by two lysines. Then, the conjugated protein was biotinylated using sortase A and assembled with tetrameric SA-4A to yield a cytotoxic tetrameric conjugate. The efficiency of conjugation, biotinylation and correctness of complex assembly were confirmed by SDS-PAGE with CBB staining (A) and western blotting with antibodies directed against FGF2 (B). Thermal denaturation of the SDS-PAGE samples was skipped to preserve the tetrameric form of proteins. C. Biotinylation of the cytotoxic conjugate was confirmed by BLI by measuring the interaction of MMAE-FGF2V and MMAE-FGF2V-Biot with streptavidin-bearing SAX2 biosensors. Association and dissociation profiles were measured. D. The cytotoxic potential of the tetrameric conjugate MMAE-FGF2v-Biot-SA-4A was evaluated in U2OS-R1 cell line. Cells were treated with MMAE-FGF2V-Biot in the presence or absence of SA-4A at various concentrations for 96 h. Then, cells viability was assessed with the Presto Blue assay. Results are mean values from three experiments +/-SEM. Fig. S3. Development o [file 12929_2021_767_MOESM1_ESM.pdf]

Supplementary data for

Modular self-assembly system for development of oligomeric, highly internalizing and potent cytotoxic conjugates targeting fibroblast growth factor receptors

Marta Poźniak[1], Natalia Porębska[1], Kamil Jastrzębski[2], Mateusz Adam Krzyścik[1], Marika Kucińska[1], Weronika Zarzycka[1], Agnieszka Barbach[1], Małgorzata Zakrzewska[1], Jacek Otlewski[1], Marta Miączyńska[2], and Łukasz Opaliński[1]*

[1]Faculty of Biotechnology, Department of Protein Engineering, University of Wroclaw, Joliot-Curie 14a, 50-383 Wroclaw, Poland

[2]Laboratory of Cell Biology, International Institute of Molecular and Cell Biology, Warsaw 02-109, Poland.

*Correspondence should be addressed to Ł.O (lukasz.opalinski@uwr.edu.pl)

Supplementary Figure Legends

Fig. S1. Internalization of various FGF1-SA oligomers via FGFR1-mediated endocytosis.

A. Live cell imaging of endocytosis of monomeric FGF1E-AviTag-Biot and FGF1E-AviTag-Biot in combination with different SA variants. U2OS-R1 cells were incubated on ice for 40 min with Alexa Fluor 488 C5 maleimide-labeled FGF1E-AviTag-Biot alone or in the presence of SA variants of different valency (from 1 to 3). Then, cells were transferred to 37°C and imaged live for 60 min using a spinning disk confocal microscope. Images taken at the indicated time points are shown. The scale bar represents 50 μm. **B.** Quantitative analysis of endocytosis of FGF1E-AviTag-Biot alone or in combination with different variants of streptavidin. Mean values from three live cell imaging experiments +/-SEM are shown.

Fig. S2. Development of the tetrameric MMAE-FGF2$_V$-Biot-SA-4A

A - B. FGF2$_V$ was conjugated to the cytotoxic compound MMAE via N-terminal cysteine flanked by two lysines. Then, the conjugated protein was biotinylated using sortase A and assembled with tetrameric SA-4A to yield a cytotoxic tetrameric conjugate. The efficiency of conjugation, biotinylation and correctness of complex assembly were confirmed by SDS-PAGE with CBB staining (A) and western blotting with antibodies directed against FGF2 (B). Thermal denaturation of the SDS-PAGE samples was skipped to preserve the tetrameric form of proteins. **C.** Biotinylation of the cytotoxic conjugate was confirmed by BLI by measuring the interaction of MMAE-FGF2$_V$ and MMAE-FGF2$_V$-Biot with streptavidin-bearing SAX2 biosensors. Association and dissociation profiles were measured. **D.** The cytotoxic potential of the tetrameric conjugate MMAE-FGF2$_V$-Biot-SA-4A was evaluated in U2OS-R1 cell line. Cells were treated with MMAE-FGF2$_V$-Biot in the presence or absence of SA-4A at various concentrations for 96 h. Then, cells viability was assessed with the Presto Blue assay. Results are mean values from three experiments +/-SEM.

Fig. S3. Development of the tetrameric MMAE-Affibody$_{HER2}$-Biot-SA-4A targeting HER2 receptor.

A. Affibody$_{HER2}$ was conjugated with cytotoxic MMAE via an N-terminal KCK motif. Then, conjugated protein was biotinylated with using sortase A and assembled with SA-4A to obtain a tetrameric MMAE-Affibody$_{HER2}$-Biot-SA-4A conjugate. The purity and identity of the proteins at each reaction step were verified by SDS-PAGE with CBB staining. To preserve the tetrameric form of the protein during SDS-PAGE, the thermal denaturation step was omitted. **B.** BLI comparison of MMAE-Affibody$_{HER2}$ and MMAE-Affibody$_{HER2}$-Biot binding to streptavidin using SAX2 biosensors. Association and dissociation profiles were measured. **C.** The cytotoxic potential of monomeric MMAE-Affibody$_{HER2}$ and tetrameric MMAE-Affibody$_{HER2}$-Biot-SA-4A was measured in the SKBR3 cell line. Cells were treated with MMAE-Affibody$_{HER2}$ or MMAE-Affibody$_{HER2}$-Biot-SA-4A at various concentrations for 96 h. Then, cell viability was assessed with the Presto Blue assay. Results are mean values from three experiments +/-SEM. 4x – monomeric MMAE-Affibody$_{HER2}$ was used at four times higher concentrations in the experiments to provide cells with equal molar concentrations of drug and targeting molecule.

Supplementary Figures

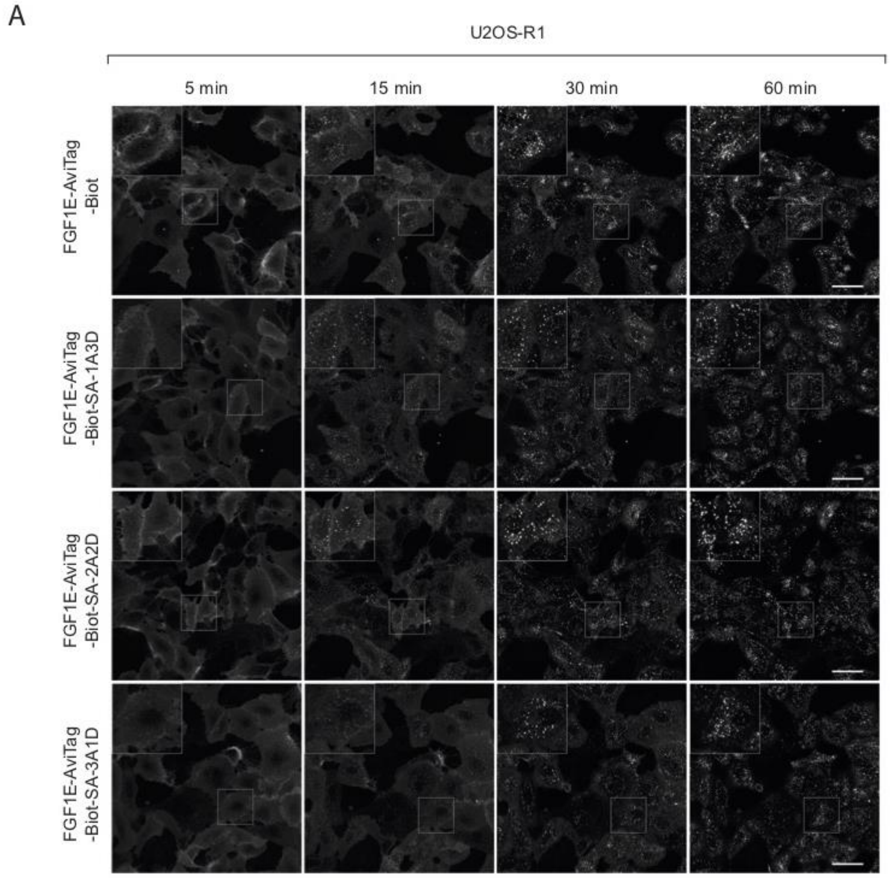

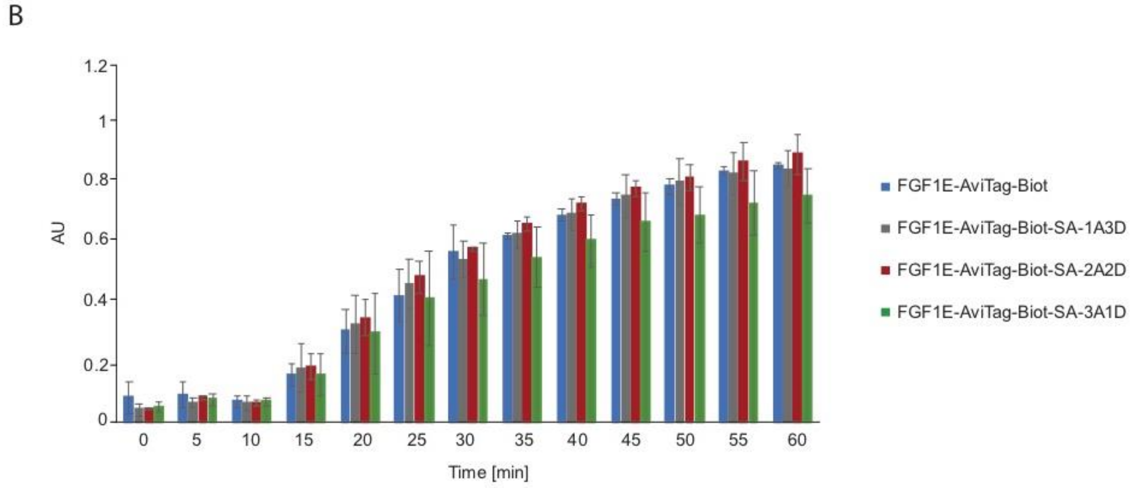

Fig. S1

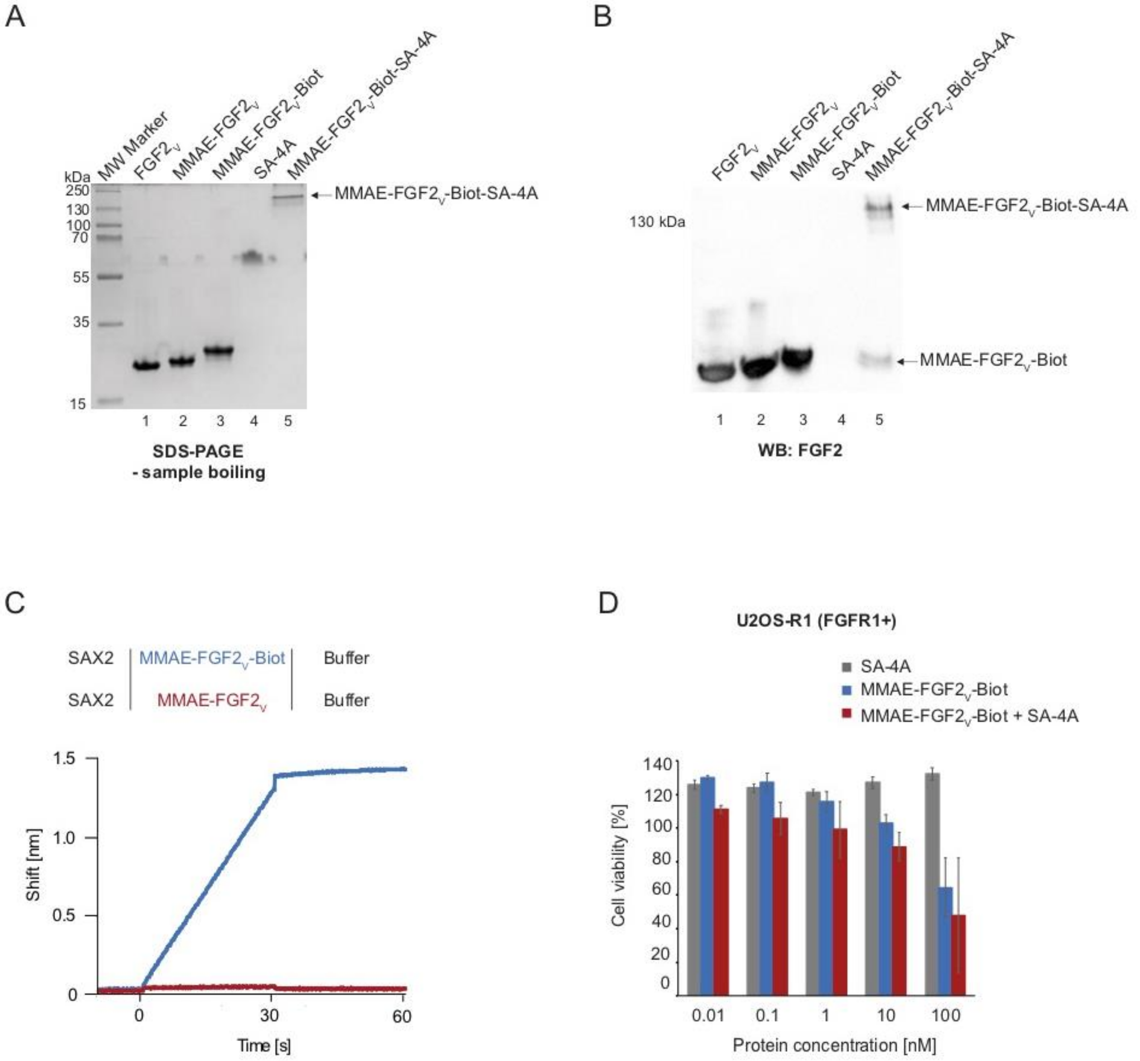
A
kDa
MW Marker
FGF2V
MMAE-FGF2V
MMAE-FGF2V-Biot
SA-4A
MMAE-FGF2V-Biot-SA-4A
250
130
100
70
55
35
15
1 2 3 4 5
SDS-PAGE
- sample boiling
B
FGF2V
MMAE-FGF2V
MMAE-FGF2V-Biot
SA-4A
MMAE-FGF2V-Biot-SA-4A
130 kDa
MMAE-FGF2V-Biot
1 2 3 4 5
WB: FGF2
C
SAX2
MMAE-FGF2V-Biot
Buffer
SAX2
MMAE-FGF2V
Buffer
Shift [nm]
1.5
1.0
0.5
0
0
30
60
Time [s]
D
U2OS-R1 (FGFR1+)
SA-4A
MMAE-FGF2V-Biot
MMAE-FGF2V-Biot + SA-4A
Cell viability [%]
140
120
100
80
60
40
20
0
0.01
0.1
1
10
100
Protein concentration [nM]

Fig. S2

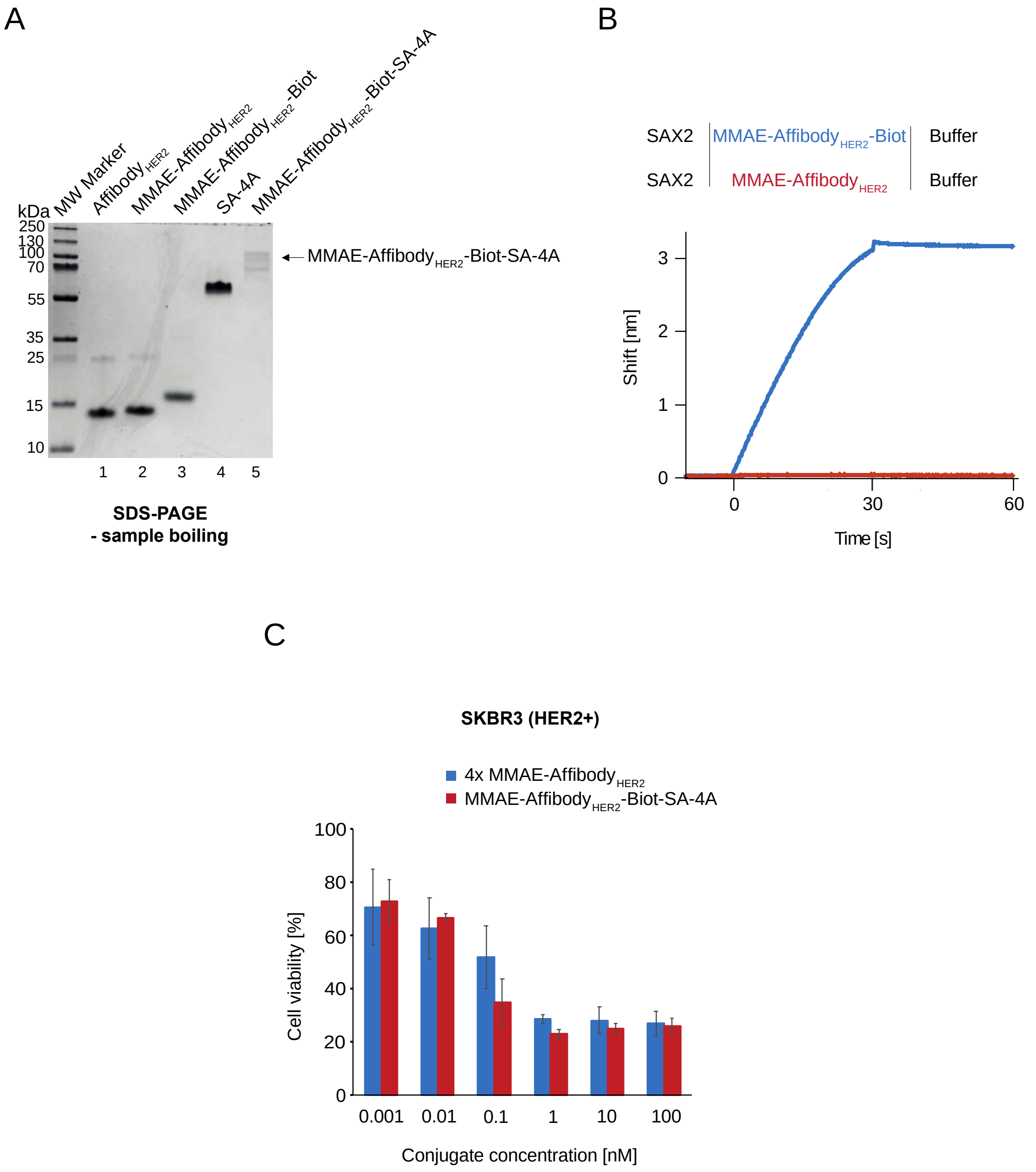
A
kDa
MW Marker
Affibody HER2
MMAE-Affibody HER2
MMAE-Affibody HER2 -Biot
SA-4A
MMAE-Affibody HER2 -Biot-SA-4A
250
130
100
70
55
35
25
15
10
MMAE-Affibody HER2 -Biot-SA-4A
1
2
3
4
5
SDS-PAGE
- sample boiling
B
SAX2
MMAE-Affibody HER2 -Biot
Buffer
SAX2
MMAE-Affibody HER2
Buffer
Shift [nm]
3
2
1
0
0
30
60
Time [s]
C
SKBR3 (HER2+)
4x MMAE-Affibody HER2
MMAE-Affibody HER2 -Biot-SA-4A
Cell viability [%]
100
80
60
40
20
0
0.001
0.01
0.1
1
10
100
Conjugate concentration [nM]

Fig. S3
